# Microglial-Derived IGF-1 Serves as a Regulator for Neuroimmune Homeostasis During Viral-Induced Demyelination

**DOI:** 10.3390/v18050550

**Published:** 2026-05-09

**Authors:** Vanessa M. Scarfone, Collin Pachow, Pauline U. Nguyen, Anita Lakatos, Jamie-Jean De La Torre, Alisa Xie, Kellie Fernandez, Charlene Collado, Kaitlin Murray, Roberto Tinoco, Craig M. Walsh, Trevor Owens, Agnieszka Wlodarczyk, Thomas E. Lane

**Affiliations:** 1 Sue & Bill Gross Stem Cell Research Center, University of California, Irvine, CA 92697, USA; vscarfon@hs.uci.edu (V.M.S.); nguyenpu@hs.uci.edu (P.U.N.); alakatos@hs.uci.edu (A.L.); cwalsh@uci.edu (C.M.W.); 2 Department of Molecular Biology and Biochemistry, School of Biological Sciences, University of California, Irvine, CA 92697, USA; collin.pachow@gmail.com (C.P.); gilmerj@uci.edu (J.-J.D.L.T.); rtinoco@uci.edu (R.T.); 3 Department of Neurobiology and Behavior, University of California, Irvine, 2213 McGaugh Hall, Irvine, CA 92697, USA; alisad1@uci.edu (A.X.); kellief@uci.edu (K.F.); charlene.r.collado@gmail.com (C.C.); kaitlimm@uci.edu (K.M.); 4 Center for Virus Research, University of California, Irvine, CA 92697, USA; 5 Neurobiology Research, Department of Molecular Medicine, University of Southern Denmark, 5000 Odense, Denmark; towens@health.sdu.dk (T.O.); awlodarczyk@health.sdu.dk (A.W.)

**Keywords:** coronaviruses, demyelination, neuroinflammation, neurovirology

## Abstract

This study investigated the role of microglia-derived insulin-like growth factor 1 (IGF-1) in modulating host defense and disease progression in a viral model of neuroinflammation and demyelination. Intracranial infection of susceptible mice with the glial-tropic JHM strain of mouse hepatitis virus (JHMV) induces acute encephalomyelitis, followed by an immune-mediated demyelinating disease that mimics many clinical and histologic features of multiple sclerosis (MS). Utilizing an inducible fractalkine receptor (*Cx3cr1*) promoter-driven Cre-loxP recombinant system, we performed timed ablation of *Igf1* in microglia to assess its impact on the central nervous system (CNS) response to JHMV. While the loss of microglial IGF-1 did not impair the control of viral replication, it significantly exacerbated spinal cord demyelination. CyTOF and imaging mass cytometry analysis of spinal cords indicated increased myelin damage was associated with increased accumulation of CD8^+^Ly6C^+^ effector T cells and reduced expression of TREM2 that impaired transition into a disease-associated microglia (DAM) phenotype capable of sensing and potentially mitigating myelin damage. Collectively, these findings argue that microglial IGF-1 is a non-redundant coordinator of the CNS immune responses that occur in response to CNS viral infection.

## 1. Introduction

Intracranial (i.c.) inoculation of susceptible mice with the neurotropic JHM strain of mouse hepatitis virus (JHMV) results in an acute encephalomyelitis characterized by widespread viral replication in glial cells, with relative sparing of neurons [1,2,3]. Innate immune responses, including expression of type I interferon and chemokines that attract myeloid cells to the CNS, are important in limiting viral replication as well as allowing virus-specific T cells access to the brain parenchyma [4]. Both CD4^+^ and CD8^+^ T cells are recruited into the CNS of JHMV-infected mice by T cell chemotactic chemokines CCL5, CXCL9 and CXCL10 and these cells control viral replication through secretion of interferon-γ (IFN-γ) and cytolytic activity, respectively [5,6,7,8,9,10,11,12]. In addition, microglia aid in host defense against JHMV infection by enhancing anti-viral responses by virus-specific CD4^+^ T cells [13,14]. Antibody-secreting cells (ASCs) are also capable of responding to CXCL9 and CXCL10 and aid in host defense by preventing viral recrudescence [15,16]. Nonetheless, sterile immunity is not achieved, and the majority of animals that survive the acute stage of disease develop immune-mediated demyelination in which both virus-specific T cells and macrophages amplify the severity of white matter damage associated with hind-limb paralysis [1,2,13,17,18]. Regulatory T cells (Tregs) can dampen the severity of JHMV-induced demyelination by restricting T cell proliferation and reducing pro-inflammatory cytokine/chemokine expression [19].

Microglia, the resident immune cell of the CNS, are a heterogeneous population of cells with various subset-dependent functions and play a prominent role in regulating CNS homeostasis as well as aiding in host defense following microbial infection. Microglia are important for baseline developmental myelination [20,21] and facilitating remyelination, in part, through clearance of myelin debris [22,23,24,25]. Within the context of JHMV infection, microglia also aid in limiting the severity of demyelination via regulating inflammatory gene expression as well as enhancing remyelination, in part, by phagocytozing myelin debris [14,26,27,28].

Insulin-like growth factor 1 (IGF-1) is an indispensable somatotropic axis growth factor crucial for growth and organ development. It is predominantly expressed by microglia and macrophages within the CNS [29] and plays a critical role in CNS development and myelination. Microglia aid oligodendrocytes, the myelinating cells of the CNS, through their production of insulin-like growth factor 1 (IGF-1), which stimulates proliferation and directs preferential differentiation of oligodendrocyte progenitor cells (OPCs) into oligodendrocytes [30,31,32,33]. Genetic deletion of *Igf1* from CD11c+ microglia results in reduced brain weight and a dramatic impairment in primary myelination arguing for unique populations of microglia required for myelogenesis in the developing CNS [34]. In addition to a role in regulating myelination, microglia-derived IGF-1 has also been implicated in modulating neuroinflammation. Genetic deletion of IGF-1R (*Igf1r*), the high-affinity receptor for IGF-1, from CNS-resident microglia and macrophages led to a more severe disease course in experimental autoimmune encephalomyelitis (EAE), the prototypic model of autoimmune-mediated neuroinflammation and demyelination, demonstrating an important role for the IGF-1:IGF-1R signaling axis in modulating immune responses within the CNS [35].

In studies presented here, we show that microglia-specific deletion of *Igf1* leads to an increase in clinical disease severity following JHMV infection that was independent of the control of viral replication within the CNS. Rather, targeted silencing of IGF-1 resulted in extensive spinal cord demyelination that correlated with worsened clinical disease severity. To further understand how the loss of microglial IGF-1 drives immunopathology, we utilized CyTOF mass cytometry and IMC to provide a high-dimensional characterization of the immune landscape within the spinal cord. This analysis revealed that the absence of microglial IGF-1 does not result in a global, non-specific influx of all immune cells, but rather a selective expansion of effector CD8^+^Ly6C^+^ T cells and a concomitant loss of regulatory T cell subsets. In addition, IGF-1 deficiency in microglia led to reduced expression of TREM2 that was inversely correlated with clinical disease severity. Based upon findings from this study, we propose a model in which microglial-derived IGF-1 serves as a critical rheostat for neuroimmune homeostasis during viral-induced demyelination. While IGF-1 is not required for the primary clearance of JHMV, its presence is important for preventing the transition from protective inflammation to chronic immunopathology.

## 2. Materials and Methods

### 2.1. Mice and Viral Infection

MG-*Igf1*^KO^ (*Cx3cr1*^CreERT/+^:*Igf1*^fl/fl^) and control mice (*Cx3cr1*^CreERT/+^:*Igf1*^+/+^) bred in the UCI vivarium and pups were genotyped at 3 weeks of age as previously described [34]. Beginning at 4–6 weeks of age, mice received 1 mg of tamoxifen (dissolved in corn oil) and animals received 100 µL per mouse via intraperitoneal (i.p.) injection, twice daily (six hours apart), for five consecutive days. Experimental mice were infected via intracranial injection [28,36] with 1200 plaque-forming units (PFUs) of JHMV suspended in 30 µL of Hank’s Balanced Salt Solution (HBSS)) at 6–8 weeks of age and clinical scores were measured, blind to experimental group, following JHMV inoculation using an established scoring system [28,36]. Experimental mice were sacrificed at days 7, 14, and 21 p.i. at which point brains and spinal cords were collected. All animal studies were reviewed and approved by the University of California, Irvine Institutional Animal Care and Use Committee (IACUC).

### 2.2. Determination of Viral Burden

To determine viral titers within brains, experimental animals were sacrificed at defined times p.i., with brains isolated and homogenized, and plaque assays performed on the DBT astrocytoma cell line as described previously [28,37]. Spinal cords of JHMV-infected controls and MG-*Igf1*^KO^ mice were homogenized, with RNA extraction, cDNA synthesis, and qPCR for comparison of JHMV mRNA levels performed as previously described [28]. Primer sequences used were: GAPDH—forward sequence, AACTTTGGCAT TGTGGAAGG; reverse sequence, GGATGCAGGGATGATGTTCT: JHMV Matrix glycoprotein—forward sequence, TCAACCCCGAAACAAACAACC; reverse sequence, GGCTGTTAGTGTATGG TAATCCTCA. qPCR was performed using the Bio-Rad iQ5 and iTaq^TM^ Universal SYBR^®^ Green Supermix (Bio-Rad, Hercules, CA, USA). Reactions were 10 µL, and the machine was set to run for 1 cycle (95 °C for 3 min), followed by 40 cycles (95 °C for 10 s, then 55 °C for 30 s). Ct values for each sample were normalized to an internal control (GAPDH), yielding the dCt values; lower dCT values indicate higher mRNA levels while higher dCT values are indicative of lower mRNA levels as more cycles of amplification are required to detect a signal.

### 2.3. Cell Isolation and Flow Cytometry

Flow cytometry was performed to identify inflammatory cells entering the CNS using established protocols [38]. In brief, single cell suspensions were generated from tissue samples by grinding with frosted microscope slides. Immune cells were enriched via a 2-step Percoll cushion (90% and 63%), and cells were collected at the interface of the two Percoll layers. Before staining with fluorescent antibodies, isolated cells were incubated with anti-CD16/32 Fc block (BD Biosciences, San Jose, CA, USA) at a 1:100 dilution. Immuno-phenotyping of cells was performed using commercially available antibodies specific for the following cell surface markers: CD4 (Invitrogen, 11-0042-82, California, CA, USA), CD8a (Invitrogen, 17-0081-82), CD11b (Abcam, Cambridge, UK Cat# ab24874) and CD45 (BioLegend, San Diego, CA, USA cat#s 103114; 103130). Cells were simultaneously incubated with LIVE/DEAD Aqua Dead Cell Stain (Thermo Fisher Scientific, Waltham, MA, USA Cat# L34966).

The following flow cytometric gating strategies were employed for inflammatory cells isolated from the CNS: macrophages/myeloid cells (CD45^hi^CD11b^+^) and microglia (CD45^lo^CD11b+); FITC-conjugated rat anti-mouse CD4 and a PE-conjugated tetramer specific for the CD4 immunodominant epitope present within the JHMV matrix (M) glycoprotein, spanning amino acids 133-147 (M133-147 tetramer), to determine total and virus-specific CD4^+^ cells [28]. APC-conjugated rat anti-mouse CD8a and a PE-conjugated tetramer specific for the CD8 immunodominant epitope present in the spike (S) glycoprotein, spanning amino acids 510-518 (S510-518), to identify total and virus-specific CD8^+^ cells. Data were collected using a Novocyte (Agilent, Santa Clara, CA, USA) flow cytometer and analyzed with FlowJo (BD Biosciences, San Jose, CA, USA) software version 10.0.8 and 11.0.2). For FACS, cells were resuspended in FACS buffer, and compensation tubes were prepared with UltraComp eBeads Compensation Beads (Thermo Fisher Scientific, Waltham, MA, USA). Microglia (CD11b^+^, CD45^lo^) and macrophages (CD11b^+^, CD45^hi^) were sorted using the BD FACSAria Fusion Flow Cytometer (BD Biosciences, San Jose, CA, USA).

### 2.4. Histology

Mice were euthanized at defined times p.i. with isoflurane, and immediately cardiac perfused with 20 mL of 1X PBS, and spinal columns were isolated and stored in 4% paraformaldehyde at 4 °C for 24–36 h. The length of spinal cord extending from thoracic vertebrae 6–10 was carefully removed and cryoprotected in 30% sucrose at 4 °C for a minimum of 3 days, then cut into 1 mm transverse blocks and processed to preserve the craniocaudal orientation, and subsequently embedded in optimum cutting temperature (OCT) compound (VWR, Radnor, PA, USA). Spinal cord tissue was coronally cryosectioned at eight microns (μm) thick. Sections were stained with hematoxylin and eosin (H&E) in combination with luxol fast blue (LFB), with between 5 and 8 sections/mouse analyzed. Areas of total white matter and demyelinated white matter were determined with Image J Software version 1.54m (NIH, Bethesda, MD, USA) and demyelination was scored as a percentage of total white matter from spinal cord sections analyzed as previously described [14,28,39,40,41].

### 2.5. Elisa

ELISA for murine myelin oligodendrocyte glycoprotein (MOG) was performed via the Abcam kits, ab282304 according to the manufacturer’s specifications. All absorbance measurements were made using the Varioskan LUX (Thermo Fisher Scientific, Waltham, MA, USA).

### 2.6. Mass Cytometry (CyTOF) Acquisition and Analysis

Antibody cocktails were prepared for surface and intracellular/nuclear targets at the concentrations indicated in Supplementary Table S1, each in a final volume of 100 µL of cell staining buffer for up to 3 × 10^6^ cells. All antibodies were provided by the UC Irvine Stem Cell Research Center Flow & Mass Cytometry Core antibody bank and conjugated using Standard BioTools (SBI) (South San Francisco, CA, USA) Maxpar X8 and MCP9 (cadmium) metal conjugation kits according to the manufacturer’s protocol. Cells isolated by Percoll gradient were stained for viability with cisplatin (SBI Viability Staining Protocol), followed by surface antibody staining, cytoplasmic/secreted protein antibody staining, fixation, and Cell-ID Intercalator-IR staining, performed sequentially according to SBI’s Maxpar Cytoplasmic/Secreted Antigen Staining with Fresh Fix protocol. Following overnight intercalator staining, cells were centrifuged (800× *g*, 5 min) and washed once with SBI Maxpar Cell Staining Buffer to remove excess intercalator, then washed twice with SBI Maxpar Cell Acquisition Solution (CAS). Pellets were resuspended in 0.1× EQ normalization bead solution (1:9 beads to CAS) and filtered through a 35 µm cell strainer immediately before acquisition at a target concentration of 1.0 × 10^6^ cells/mL (300–500 events/s). The SBI Helios was tuned prior to analysis using CyTOF Tuning Solution. All FCS files were normalized together using SBI CyTOF Software v7.0.8493. Normalized FCS files were analyzed in OMIQ (Dotmatics www.omiq.ai (accessed on 30 March)). Batch effects across acquisition runs were corrected using CytoNorm [42]. Standard cleanup gating was applied to exclude normalization beads, doublets, dead cells, and debris. Unsupervised clustering was performed using FlowSOM [43] with 25 metaclusters, of which 23 represented immune lineages; dimensionality reduction was visualized by t-SNE, with FlowSOM metacluster identities overlaid. Metacluster phenotypes were annotated based on median marker expression profiles (Supplementary Figure S2). Differential abundance analysis was performed by calculating the frequency of each metacluster as a percentage of total CD45^+^ cells per mouse. Genotype comparisons were assessed by two-tailed Mann–Whitney U test on mouse-level cluster frequencies (*n* = 4 per group). To characterize coordinated immune cell behavior, Pearson correlation coefficients were computed on metacluster frequencies separately within Cre-CTRL and MG-*Igf1*^KO^ groups. A subset of functionally relevant populations was selected for correlation analysis; correlations with |*r*| > 0.90 were considered strong. *p*-values were corrected for multiple comparisons.

### 2.7. Statistical Analysis

Statistical significance for the survival curve was assessed by log-rank (Mantel–Cox) test, with significance defined as *p* < 0.05. Clinical scores were analyzed as previously described [28,36,44]; *** *p* < 0.001. Viral plaque-forming units and qPCR ΔCt values were compared using two-tailed, unpaired *t*-tests. MOG concentrations were compared by one-way ANOVA with Tukey’s multiple comparisons test, and demyelination percentages (C) by two-way ANOVA with Šídák’s multiple comparisons test. Remaining group comparisons used multiple unpaired *t*-tests without correction for multiple comparisons. All IMC statistical analyses were performed using Python (v3.13.5) with SciPy (v1.15.3), NumPy (v2.1.3), and statsmodels (v0.14.4) unless otherwise noted. CyTOF differential abundance analysis and visualization were performed in OMIQ (Dotmatics) and GraphPad Prism (10.6.1).

Threshold robustness. Percentile-based thresholding (75th percentile of arcsinh-transformed intensities) and hierarchical cell-type assignment are standard for IMC data. We confirmed that cell-type abundances scaled monotonically with threshold stringency and that the direction and magnitude of key findings (Act. MG-DAM, Act. MG-CD8 spatial enrichment, and their inverse correlation with TREM2 expression) were consistent across the p70–p85 range, supporting the robustness of our analytical pipeline.

Group comparisons. Two-group comparisons of mouse-level summary statistics (cluster frequencies, median marker intensities, enrichment z-scores, DAM maturation ratios) were assessed by two-tailed Mann–Whitney U tests (scipy.stats.mannwhitneyu), appropriate for small sample sizes (CyTOF: *n* = 4 per group; IMC: *n* = 6 per group) without assumption of normality. Effect sizes are reported as rank-biserial correlation (*r* = 1 − 2U/n_1_n_2_), with |*r*| ≥ 0.5 considered large, |*r*| ≥ 0.3 medium, and |*r*| < 0.3 small.

Multiple comparison correction. For IMC mouse-level group comparisons, *p*-values across 8 tests were corrected using the Benjamini–Hochberg false discovery rate (FDR) procedure at Q = 0.05 (statsmodels.stats.multitest.multipletests, method = “fdr_bh”). The 8-test family comprised: TREM2 intensity, DAM maturation ratio, activated microglia self-clustering, activated microglia–CD8 T cell proximity, astrocyte–neuron enrichment, neuron–other enrichment, CD163^+^–CD8 T cell enrichment, and astrocyte–CD4 T cell enrichment. Both raw and FDR-adjusted *p*-values are reported; adjusted *p*-values < 0.05 were considered significant. For TREM2–spatial Spearman correlations a separate 4-test FDR family was applied. CyTOF differential abundance *p*-values across 23 metaclusters are reported uncorrected due to the exploratory nature of testing at *n* = 4 per group.

Correlation analyses. Spearman rank correlations (scipy.stats.spearmanr) were used to assess monotonic relationships between continuous variables, including TREM2 intensity versus spatial enrichment z-scores (all 12 animals) and spatial metrics versus clinical scores (MG-*Igf1*^KO^ only, *n* = 6). Within-genotype clinical–spatial correlations are reported with uncorrected *p*-values and effect sizes (ρ^2^ as variance explained) due to limited statistical power at *n* = 6; these analyses did not survive FDR correction (10-test family). CyTOF cluster–cluster correlations were assessed by Pearson correlation on cluster frequencies within each genotype group separately; correlations with |*r*| > 0.90 were considered strong.

Spatial enrichment analysis. Permutation-based neighborhood enrichment was computed for each tissue image using the *N* × *N* method (Squidpy framework). Nine exclusive cell types were assigned using percentile-based thresholds (75th percentile for structural myeloid, and T cell markers) on arcsinh-transformed (cofactor = 1) marker intensities. Spatial neighbor graphs were constructed using scipy.spatial.cKDTree with a 30-pixel (30 µm) interaction radius. For each cell-type pair, observed neighbor frequencies were compared to null distributions generated by 500 random permutations of cell-type labels, yielding enrichment z-scores per image. Mouse-level z-scores were obtained by averaging across images per animal (images with <50 cells excluded). The primary analysis used random seed = 42.

Cell-level analyses. Arg1 expression on Iba1^+^CD163^+^ cells (15,979 cells) stratified by proximity to CD8 T cells (near: ≤30 pixels; far: >30 pixels) was compared by Mann–Whitney U test at the cell level. Cell-level *p*-values reflect the large sample size and are not included in the mouse-level FDR family; they should be interpreted alongside mouse-level comparisons and effect sizes.

IMC image processing. IMC data were acquired on a Hyperion imaging system (Standard BioTools) using a 27-marker panel across 39 tissue images from 12 animals (6 Cre-CTRL, 6 MG-*Igf1*^KO^) at 21 days post-JMHV infection. Image denoising was performed using DIMR (IMC Denoise). Cell segmentation was performed using Cellpose-SAM (cpsam model, v4.0). Marker intensities were arcsinh-transformed with cofactor = 1. A total of 62,204 immune-associated cells were retained from approximately 184,000 segmented objects.

Software. CyTOF data were processed in OMIQ (Dotmatics) byunsupervised clustering by FlowSOM [43]. IMC spatial analysis was implemented in Python using scipy.spatial.cKDTree for neighbor graph construction and custom permutation code for enrichment testing. Figures were generated using matplotlib (v3.x). All analysis code is available by contacting corresponding authors.

### 2.8. Imaging Mass Cytometry

#### 2.8.1. Tissue Preparation and Antibody Staining

Spinal cord tissue was sectioned at 10 µm and mounted on glass microscope slides. Sections were desiccated for a minimum of 6 h prior to antigen retrieval. Heat-induced epitope retrieval was performed in 1 mM sodium citrate buffer (pH 6.0) at 95–100 °C for 20 min, after which slides were allowed to cool to approximately 70 °C. Following rinses in PBS, sections were blocked with 3% BSA for 30 min. A 36-marker metal-conjugated antibody panel (Supplementary Table S1) was prepared as a cocktail at indicated concentrations in a final volume of 125 µL per slide. All slides (*n* = 20 from 12 animals) were stained simultaneously in a single batch to eliminate inter-batch variability. Sections were incubated with the antibody cocktail overnight at 4 °C. Following antibody incubation, slides were counterstained with iridium intercalator (Ir191/193, 1:400, 30 min) for nuclear identification, washed, and air-dried prior to acquisition.

#### 2.8.2. IMC Acquisition

Imaging mass cytometry was performed on a Hyperion Imaging System (Standard BioTools, formerly Fluidigm). Regions of interest (ROIs) were selected to encompass entire spinal cord cross-sections. A total of 39 tissue images were acquired from Cre-CTRL (*n* = 6) and MG-*Igf1*^KO^ (*n* = 6) mice at 21 days post-JHMV infection. Acquisition was performed at 200 Hz laser frequency, ablation energy 6, and 1 µm pixel resolution.

#### 2.8.3. Image Denoising

Raw IMC images were denoised using the IMC-Denoise pipeline [45]. For each marker channel, 30,000 training patches were generated and used to train sequential DIMR (hot pixel removal) and DeepSNiF (shot noise filtering) models [45]. Denoising was performed on the UCI HPC3 computing cluster. Quality control included visual inspection of denoised versus raw images for each marker. All downstream analyses were performed on denoised images.

#### 2.8.4. Cell Segmentation and Marker Quantification

Cell segmentation was performed using Cellpose-SAM (cpsam model, v4.0 www.cellpose.org (accessed on 30 March)); with GPU acceleration. A multi-pass strategy was employed to capture morphologically distinct cell populations. Pass 1 targeted large nuclei (estimated diameter = 16 pixels, flow_threshold = 0.3, cellprob_threshold = 0.0) and Pass 2 targeted small immune cells (estimated diameter = 6 pixels, flow_threshold = 0.4, cellprob_threshold = −1.0). Both passes used a two-channel input consisting of Ir191 and Ir193 DNA intercalator channels, each normalized to the 1st–99.8th percentile range. Segmentation masks from Pass 2 were merged with Pass 1 results; new cells were added only if they overlapped less than 50% with existing segmentations. Cells exceeding 500 px^2^ were excluded as likely doublets or debris. Mean pixel intensity across all 36 marker channels was extracted for each segmented cell using scikit-image regionprops. This yielded approximately 184,000 segmented objects across 39 tissue images at 1.0 µm pixel resolution.

#### 2.8.5. Data Transformation

Raw mean pixel intensities were arcsinh-transformed (cofactor = 1; i.e., arcsinh(x)) prior to cell-type assignment and all downstream analyses. This transformation compresses the dynamic range of IMC signal intensities while preserving rank order, enabling percentile-based gating across markers with varying expression scales.

#### 2.8.6. Cell-Type Assignment

Nine exclusive cell types were assigned using percentile-based thresholds on arcsinh-transformed marker intensities. Structural markers (GFAP for astrocytes, Map2 for neurons, Olig2 for oligodendrocytes) and myeloid markers (Iba1, CD68, TREM2, CD163) were thresholded at the 75th percentile, while T cell markers (CD3e/CD8a for CD8 T cells; CD4/CD45 for CD4 T cells) were thresholded at the 75th percentile. Cell types were assigned hierarchically in the following order: astrocyte (GFAP^+^), neuron (Map2^+^), oligodendrocyte (Olig2^+^), microglia (Iba1^+^), activated microglia (Iba1^+^CD68^+^), DAM (Iba1^+^CD68^+^TREM2^+^), CD4 T cell (CD4^+^CD45^+^), and CD8 T cell (CD3e^+^CD8a^+^), with later assignments overwriting earlier ones to ensure mutual exclusivity. Cells not meeting any threshold were classified as “Other.” Iba1^+^CD163^+^ regulatory myeloid cells were analyzed separately as a non-exclusive overlay population, as CD163 positivity crosses the 9-type classification boundaries. This approach yielded 62,204 immune-associated cells from approximately 184,000 segmented objects across 39 tissue images.

#### 2.8.7. Spatial Neighborhood Enrichment Analysis

Spatial relationships between cell types were quantified using permutation-tested neighborhood enrichment analysis. For each tissue image, a spatial neighbor graph was constructed by identifying all cell pairs within a 30-pixel (30 µm) interaction radius using a k-d tree algorithm (scipy.spatial.cKDTree). Observed frequencies of cell-type neighbor pairs were compared to null distributions generated by 500 random permutations of cell-type labels, yielding a z-score matrix for each pairwise interaction. Images with fewer than 50 cells were excluded. Mouse-level enrichment z-scores were obtained by averaging image-level z-scores (mean of non-NaN values) across all images per animal. Genotype comparisons were performed using two-tailed Mann–Whitney U tests (scipy.stats.mannwhitneyu, alternative = “two-sided”) on mouse-level z-scores (*n* = 6 per group), with *p*-values corrected using Benjamini–Hochberg FDR (family size = 8). The primary analysis was performed with random seed = 42.

#### 2.8.8. TREM2 and DAM Maturation Analysis

Activated microglia were defined as Iba1^+^CD68^+^ cells using the 75th percentile thresholds described above. Disease-associated microglia (DAM) were further defined as Iba1^+^CD68^+^TREM2^+^ cells. For each mouse, median TREM2 intensity was computed across all activated microglia (including DAM). The DAM maturation ratio was defined as the proportion of activated microglia meeting the TREM2 threshold (i.e., DAM/[Act_MG ^+^ DAM]). Genotype comparisons were assessed by two-tailed Mann–Whitney U test on mouse-level values (*n* = 6 per group), with *p*-values corrected as part of the 8-test BH FDR family described in Section 2.7.

#### 2.8.9. Arg1 Proximity Analysis at the Myeloid–T Cell Interface

To assess functional capacity at the myeloid–T cell interface, Arg1 expression was compared on Iba1^+^CD163^+^ cells stratified by spatial proximity to CD8 T cells. Cells within 30 pixels (30 µm) of a CD8 T cell were classified as “near” and those beyond 30 pixels as “far.” Arg1 intensity comparisons were performed at the cell level by Mann–Whitney U test across 15,979 CD163^+^ cells. Cell-level *p*-values are not included in the mouse-level FDR family and should be interpreted alongside effect sizes and mouse-level comparisons. To confirm that observed differences were specific to the spatial interface, bulk mouse-level median expression of 12 functional markers on CD163^+^ cells was compared across genotypes by Mann–Whitney U test.

#### 2.8.10. Clinical–Spatial Correlations

Within MG-*Igf1*^KO^ animals (*n* = 6), mouse-level neighborhood enrichment z-scores and TREM2 median intensity were correlated with peak clinical score and cumulative clinical burden using Spearman rank correlation. These within-genotype correlations (10-test family: 5 spatial metrics × 2 clinical scores) did not survive BH FDR correction (all *p*_adj > 0.10) and are reported with uncorrected *p*-values, effect sizes (ρ), and variance explained (ρ^2^).

## 3. Results

### 3.1. Target Ablation of Microglia IGF-1 Does Not Impair Immune-Mediated Control of Viral Replication

**Figure 1:**
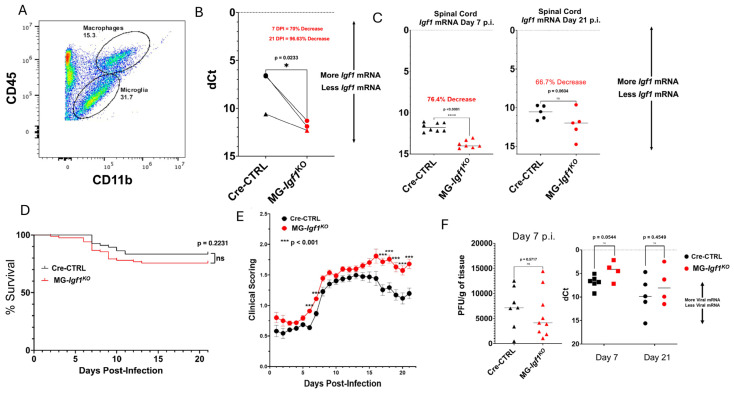
**MG-*Igf 1*^KO^ mice experience worsened clinical disease following JHMV infection.** (**A**) Representative FACS plot depicting the gating for sorting microglia based on CD45^lo^ and CD11b^+^ expression. (**B**) q-PCR analysis of RNA isolated from CD45^lo^ CD11b^+^ microglia flow sorted from brains of tamoxifen-treated Cre-CTRL and MG-*Igf1* ^KO^ mice showing deltaCt (dCt) values, representing the difference between *Igf1* mRNA expression and *Gapdh* mRNA expression in sorted microglia. Data represent 3 separate cohorts of mice from each experimental group with cohorts of mice connected via solid lines. Y-axes are reversed to reflect increases and decreases signified by upward and downward arrows, respectively. Triangles represent values collected from day 7 p.i. brains (Cre-CTRL, *n* = 7; MG-*Igf1* ^KO^, *n* = 3) while circles represent values collected from day 21 p.i. (Cre-CTRL, *n* = 3; MG-*Igf1* ^KO^, *n* = 10). (**C**) qPCR analysis of whole spinal cords from JHMV-infected tamoxifen-treated experimental mice at day 7 p.i. (Cre-CTRL, *n* = 8; MG-*Igf1* ^KO^, *n* = 8) and day 21 p.i. (Cre-CTRL, *n* = 5; MG-*Igf1* ^KO^, *n* = 5); median line is shown. For panels (**A**–**C**), statistical significances were calculated via one-tailed, unpaired *t*-tests, with significance indicated at *p*-values < 0.05. (**D**) Kaplan–Meier survival analysis of JHMV-infected Cre-CTRL mice (*n* = 66) and MG-*Igf1* ^KO^ mice (*n* = 88) shows no significant difference in survival probability through day 21 p.i.; median line is shown for both groups. (**E**) JHMV-infected MG-*Igf1* ^KO^ mice displayed increased clinical disease compared to Cre-CTRL mice at days 6, 7, and between 17 and 21 p.i.; error bars denote the standard error of the mean (SEM). (**F**) Viral burden within brains of infected mice at day 7 p.i. was determined by plaque assays for JHMV plaque-forming units (PFUs) or qPCR for JHMV membrane protein transcripts expressed within the spinal cords at days 14 and 21 p.i.; dCt values represent the difference between JHMV membrane protein mRNA expression and *Gapdh* mRNA expression in RNA extracted from whole spinal cords. Median line is shown for all groups. Statistical significance for the survival curve (**A**) was calculated via a log-rank (Mantel–Cox) test, with significance indicated at *p*-values < 0.05. Statistical significances for the clinical scoring (**B**) were calculated as previously described; *** *p* ≤ 0.001, * *p* ≤ 0.05, **** *p* < 0.0001 [28,36,44]. Statistical significances for the viral plaque-forming units and qPCR dCt values (**C**) were calculated via two-tailed, unpaired *t*-tests.

To investigate how IGF-1 signaling in microglia impacts host defense and disease in JHMV-infected mice, we employed a recently described mouse model in which microglia-specific *Igf1* is ablated through crossing CXCR1^CreER^ mice with transgenic mice carrying loxP sites flanking exon 4 of the *Igf1* gene [46]. The resulting offspring *Cx3cr1*^CreERT/2^:*Igf1^fl/fl^* mice (hereafter referred to as MG-*Igf* ^KO^ mice) was used as a tool to delete IGF-1 from microglia. MG-*Igf* ^KO^ mice and *Cx3cr1*^Cre^ were treated with tamoxifen to induce recombination and silencing of *Igf1* expression. Microglia and macrophages were FACS-sorted (Figure 1A) from pooled mouse brains of tamoxifen-treated JHMV-infected mice at days 7 and 21 p.i. and *Igf1* expression determined via qPCR. Tamoxifen treatment of MG-*Igf* ^KO^ mice resulted in a 70% decrease in *Igf1* transcripts at day 7 p.i. and by day 21 p.i., there was a 96% decrease in transcripts compared to control mice (Figure 1B). Expression of *Igf1* transcripts in whole spinal cords of JHMV-infected Cre-CTRL and MG-*Igf1* ^KO^ mice showed decreased expression at days 7 p.i. (*p* < 0.0001) and 21 p.i. in MG-*Igf* ^KO^ mice compared to Cre-CTRL mice (Figure 1C).

Targeting *Igf1* expression did not result in increased mortality between JHMV-infected MG-*Igf* ^KO^ mice compared to controls (Figure 1D); however, infected MG-*Igf1* ^KO^ mice did show increased clinical disease severity over time as compared to infected control mice with significant (*p* < 0.001) differences appearing during both acute and chronic infection (Figure 1E). The increase in clinical disease severity was not related to impaired control of viral replication as there were no differences in viral titers/RNA within the brains and spinal cords at days 7 and 21 in infected experimental mice (Figure 1F). These findings demonstrate that microglia-derived IGF-1 does not play a critical role in immune-mediated control of JHMV replication within the CNS.

### 3.2. Microglia-Derived IGF-1 Influences the Severity of Spinal Cord Demyelination in JHMV-Infected Mice

**Figure 2:**
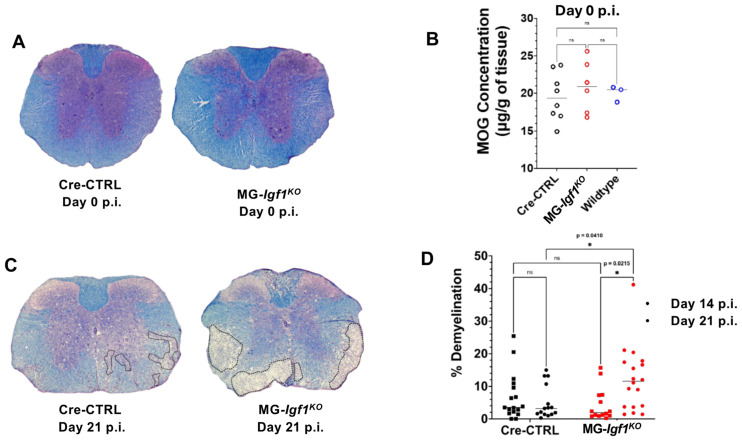
**JHMV-infected MG-*Igf1* ^KO^ mice exhibit increased spinal cord demyelination compared to Cre-CTRL mice.** Representative spinal cord sections from LFB-/H&E-stained (**A**) uninfected and (**C**) JHMV-infected spinal cords at day 21 p.i. (**B**) ELISA assay determined baseline concentrations of MOG protein from spinal cords of age-matched uninfected Cre-CTRL (black circles), MG-*Igf1* ^KO^ (red circles) mice and wildtype (WT) were not significantly different; median line is shown for all groups. (**D**) ImageJ analysis of LFB-/H&E-stained coronal spinal cord sections shows no difference in severity of demyelination between infected Cre-CTRL (black circles) and MG-*Igf1* ^KO^ (red circles) mice at day 14 p.i. yet by day 21 p.i., infected MG-*Igf1* ^KO^ mice exhibited increased (*p* ≤ 0.05) demyelination compared to infected Cre-CTRL mice. Areas of demyelination are outlined in black dashed lines. Statistical significances for the MOG concentrations were calculated via a one-way ANOVA with Tukey’s multiple comparisons test. Statistical significances for the demyelination percentages (**C**) were calculated via a two-way ANOVA with Šídák’s multiple comparisons test. * *p* ≤ 0.05.

Spinal cords were removed from uninfected and JHMV-infected MG-*Igf* ^KO^ and control mice at day 21 p.i. to evaluate the severity of demyelination. LFB staining of spinal cords from uninfected Cre-CTRL and MG-*Igf* ^KO^ mice shows no differences in myelin staining compared to uninfected control mice, indicating that tamoxifen-induced targeting of *Igf1* did not affect myelin levels in the spinal cords of adult mice (Figure 2A). This was further supported by myelin oligodendrocyte glycoprotein (MOG) ELISAs performed on uninfected spinal cords from Cre-CTRL, MG-*Igf* ^KO^, and wildtype (WT) mice showing no differences in expression levels of this myelin-associated protein (Figure 2B). Examination of spinal cords of JHMV-infected animals revealed similar levels of myelin damage at day 14 p.i. yet by day 21 p.i., there was a significant (*p* < 0.04) increase in demyelination in MG-*Igf* ^KO^ mice compared to Cre-CTRL mice (Figure 2C,D). These findings support a role for microglia-derived IGF-1 in influencing the severity of spinal cord demyelination in response to JHMV infection.

### 3.3. Increased CD8^+^ T Cell Infiltration into Spinal Cords of JHMV-Infected MG-Igf ^KO^ Mice

**Figure 3:**
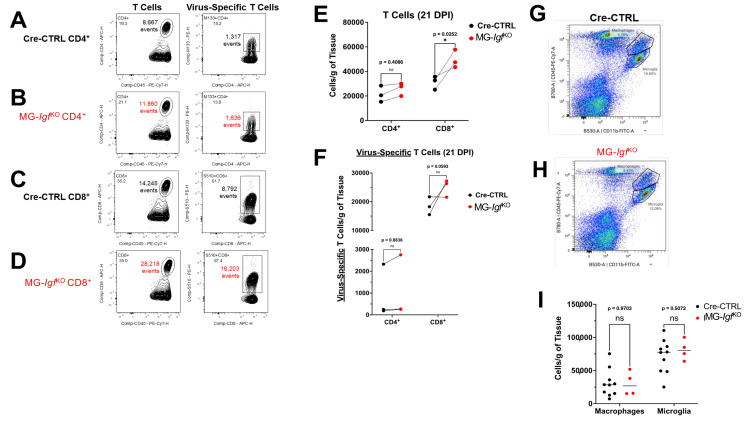
**CD8^+^ T cell infiltration is increased in spinal cords of JHMV-infected MG-*Igf1* ^KO^ mice.** Representative flow cytometric staining is shown for total CD4^+^ and CD8^+^ T cell subsets and virus-specific CD8^+^ T cells (S510-518 tetramer) and virus-specific CD4^+^ T cells (M133-147 tetramer) from spinal cords of JHMV-infected Cre-CTRL mice (black dots) (**A**,**C**) and MG-*Igf1* ^KO^ (red dots) mice (**B**,**D**) at day 21 p.i. (**E**) Quantification of flow data indicates a significant increase (*p* ≤ 0.02) in total CD8^+^ T cell numbers, but not total CD4^+^ T cells, in JHMV-infected MG-*Igf1* ^KO^ mice (*n* = 4) compared to Cre-CTRL (*n* = 10) tissue. (**F**) There were no differences in numbers of virus-specific CD4+ or CD8+ T cells between infected Cre-CTRL or MG-*Igf1* ^KO^ mice. Representative flow cytometric staining for macrophages (CD45^hi^, CD11b^+^) and microglia (CD45^lo^, CD11b^+^) from JHMV-infected spinal cords from (**G**) Cre-CTRL and (**H**) MG-*Igf1* ^KO^ mice at day 21 p.i. (**I**) Quantification of macrophage and microglia reveals no significant differences in numbers between infected Cre-CTRL (*n* = 10) and MG-*Igf1* ^KO^ mice (*n* = 4); median line is shown for all groups. Statistical significances were calculated via multiple unpaired *t*-tests and not corrected for multiple comparisons. * *p* ≤ 0.05.

Previous studies have determined that T cell infiltration into spinal cords of JHMV-infected mice amplifies white matter damage [5,44,47,48,49,50]. To determine the frequencies and numbers of total and virus-specific CD4^+^ (Figure 3A,B) and CD8^+^ T cells (Figure 3C,D), flow cytometry was performed on spinal cords of JHMV-infected MG-*Igf* ^KO^ and control mice at day 21 p.i. Quantification of T cells indicated a significant increase (*p* ≤ 0.02) in the number of total CD8^+^, but not CD4^+^ T cells, at this time point (Figure 3E). There was a trending increase in virus-specific CD8^+^ T cells, although this was not statistically significant when compared to control mice (Figure 3F). There were no differences in numbers of virus-specific CD4^+^ T cells between infected MG-*Igf* ^KO^ and control mice (Figure 3F). In addition to T cells, both macrophages and microglia can impact the severity of JHMV-induced demyelination [5,44,51,52,53]. Flow cytometric analysis of spinal cords of infected MG-*Igf*^KO^ and control mice showed no differences in numbers of either macrophages (CD45^hi^, CD11b^+^) or microglia (CD45^lo^, CD11b^+^) (Figure 3G–I).

### 3.4. CyTOF Mass Cytometry Reveals Altered T Cell Composition in Spinal Cords of JHMV-Infected MG-Igf1^KO^ Mice

**Figure 4:**
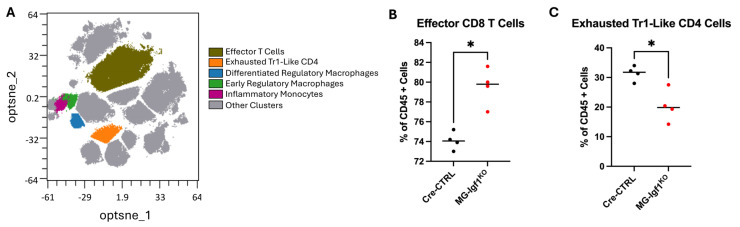
**CyTOF analysis of spinal cord immune populations following JHMV infection reveals altered T cell homeostasis in microglial IGF-1 knockout mice.** (**A**) Opt-SNE visualization of CyTOF data from JHMV-infected spinal cord at day 21 p.i. FlowSOM clustering identified [20] distinct immune populations from live CD45^+^ cells. Five key populations are highlighted: effector T cells (CD8^+^Ly6C^+^), exhausted Tr1-like CD4^+^, differentiated regulatory macrophages, early regulatory macrophages, and inflammatory monocytes. Remaining clusters shown in gray. Representative plot from MG-*Igf1*^KO^ group shown; cluster topology was equivalent between genotypes. (**B**) Differential abundance of effector CD8^+^ T cells (**left**) and (**C**) exhausted Tr1-like CD4^+^ cells (**right**) between Cre-CTRL and MG-*Igf1*^KO^ mice, expressed as percentage of CD45^+^ cells. Each point represents an individual animal. Horizontal lines indicate group means. * *p* < 0.05, unpaired two-tailed *t*-test. *n* = 4 per group.

To characterize how microglial IGF-1 deletion affects the immune landscape of the spinal cord during JHMV-induced demyelination, we performed CyTOF mass cytometry on spinal cord tissues from MG-*Igf1*^KO^ and Cre-CTRL mice. After dimensionality reduction using opt-SNE (Supplementary Figure S2), we performed unsupervised clustering using FlowSOM [42], which identified 25 metaclusters, of which 23 represented immune lineages including microglia, monocyte-derived macrophages, T cell subsets, B cells, and NK cells; two non-immune clusters were excluded from further analysis (Figure 4A, Supplementary Figures S2 and S3, and Supplementary Table S1). Differential abundance analysis of cluster frequencies between Cre-CTRL and MG-*Igf1*^KO^ mice revealed alterations in two T cell populations. Effector CD8^+^Ly6C^+^ T cells were significantly increased in MG-*Igf1*^KO^ mice compared to controls (*p* = 0.0286, Mann–Whitney U test; Figure 4B and Supplementary Table S2), consistent with prior flow cytometric findings and suggesting that loss of microglial IGF-1 permits expansion of cytotoxic effector T cells within the inflamed spinal cord. Unsupervised clustering also identified a CD4^+^ T cell subset with a Tr1-like phenotype, characterized by moderate expression of CD44, CD39, CD11a, and CD38 (hereafter “Tr1-like”). While definitive Tr1 identification requires co-expression of CD49b and LAG-3 [54], which were not included in our panel, the regulatory identity of this cluster is supported by its expression of CD39, an ectonucleotidase associated with Tr1-mediated suppression, and its strong inverse correlation with effector T cell expansion. Tr1-like CD4^+^ T cells were significantly decreased in MG-*Igf1*^KO^ mice (*p* = 0.0286, Mann–Whitney U test; Figure 4C and Supplementary Table S2). No other clusters reached statistical significance, indicating that the effects of microglial IGF-1 deletion on immune cell composition are selective rather than global.

### 3.5. Cluster Abundance Changes Reveal Disrupted Myeloid–T Cell Coordination

**Figure 5:**
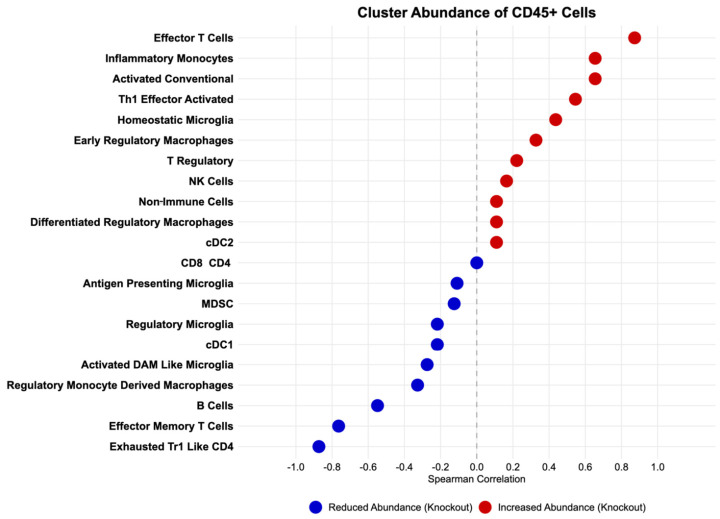
**Differential cluster abundance in MG-*Igf1*^KO^ spinal cord by CyTOF mass cytometry.** Spearman rank correlation coefficients (ρ) between cluster frequency (% of CD45^+^ cells) and genotype (Cre-CTRL = 0, MG-*Igf1*^KO^ = 1) for FlowSOM metaclusters. Positive ρ (red) indicates increased abundance in knockout (MG-*Igf1*^KO^) mice; negative ρ (blue) indicates reduced abundance. Clusters are ranked by correlation coefficient. Two non-immune metaclusters were excluded. Effector T cells and inflammatory monocytes showed the strongest positive associations with MG-*Igf1*^KO^ genotype, while exhausted Tr1-like CD4^+^ and effector memory T cells showed the strongest negative associations. *n* = 4 per group. Spearman correlations are descriptive; individual cluster significance testing is reported in Figure 4B,C.

To investigate coordinated immune cell behavior at the population level, we examined how cluster abundances shifted between Cre-CTRL and MG-*Igf1*^KO^ mice. Several immune populations showed strong directional shifts in MG-*Igf1*^KO^ spinal cords (Figure 5). Effector T cells, inflammatory monocytes, activated conventional T cells, Th1 effector activated cells, and homeostatic microglia were enriched in MG-*Igf1*^KO^ mice, while exhausted TR1-like CD4^+^ T cells, effector memory T cells, B cells, and regulatory monocyte-derived macrophages were reduced. Notably, the populations expanding in MG-*Igf1*^KO^ spinal cords were predominantly inflammatory effectors (effector T cells, Th1 effector, inflammatory monocytes), while the contracting populations were enriched for regulatory and memory phenotypes (Tr1-like CD4^+^, effector memory T cells, regulatory monocyte-derived macrophages). This coordinated divergence, concurrent expansion of effector populations alongside contraction of regulatory and memory compartments, suggests that microglial IGF1-1 loss disrupts the balance between immune activation and resolutions consistent with the spatial and functional dysregulation observed in subsequent analyses.

### 3.6. IGF-1 Deletion Impairs TREM2 Upregulation on Activated Microglia

**Figure 6:**
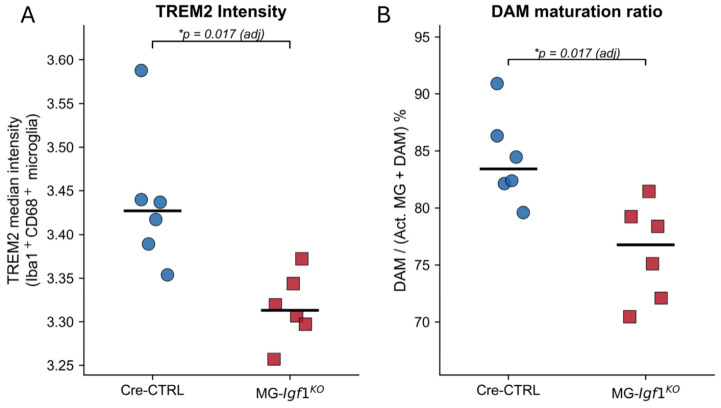
**Microglial IGF-1 deletion impairs TREM2 upregulation and DAM maturation.** (**A**) Mouse-level median TREM2 intensity on activated microglia (Iba1^+^CD68^+^) in Cre-CTRL (blue circles, *n* = 6) and MG-*Igf1*^KO^ (red squares, *n* = 6) animals at 21 days post-JHMV infection. (**B**) DAM maturation ratio, defined as DAM (Iba1^+^CD68^+^TREM2^+^)/total activated microglia (Iba1^+^CD68^+^), expressed as percentage. Black bars indicate group medians. Thresholds for marker positivity were set at the 75th percentile of arcsinh-transformed intensities across all 62,204 immune-associated cells. * *p*_adj = 0.017; two-tailed Mann–Whitney U test with Benjamini–Hochberg FDR correction (family size = 8).

To assess microglial maturation in the context of IGF-1 loss, we performed imaging mass cytometry (IMC) on demyelinating lesions from Cre-CTRL and MG-*Igf1*^KO^ mice at 21 days post-JHMV infection. A 27-marker panel (Supplemental Table S3) was applied across 39 tissue images, yielding 62,204 immune-associated cells from approximately 184,000 segmented objects. Activated microglia were identified as Iba1^+^CD68^+^ cells using 75th percentile thresholds on arcsinh-transformed marker intensities. Mouse-level median TREM2 intensity on activated microglia was significantly reduced in MG-*Igf1*^KO^ animals compared to Cre-CTRL (*p*_adj = 0.017, Mann–Whitney U test, BH FDR; Figure 6A), representing the strongest individual marker-level genotype effect in the dataset. Correspondingly, the DAM maturation ratio—defined as disease-associated microglia (Iba1^+^CD68^+^TREM2^+^) as a proportion of all activated microglia (Iba1^+^CD68^+^)—declined from 83.4% in Cre-CTRL to 76.7% in MG-*Igf1*^KO^ (*p*_adj = 0.017; Figure 6B). The identical rank-ordering of mice across both metrics (Spearman ρ = −0.821, *p* = 0.001) confirms that reduced TREM2 expression translates directly to impaired DAM conversion. Together, these findings indicate that microglial IGF-1 signaling is required for full TREM2 upregulation during the activated-to-DAM transition in viral demyelination, consistent with a maturation block at the level of the damage-sensing program.

### 3.7. Spatial Immune Niche Reorganization in MG-Igf1^KO^ Lesions

To determine whether IGF-1 loss alters the spatial organization of immune cells within demyelinating lesions, we performed permutation-tested neighborhood enrichment analysis. Nine exclusive cell types were assigned using percentile-based thresholds (75th percentile for all markers). For each tissue image, spatial neighbor graphs were constructed using a 30-pixel (30 µm) interaction radius, and observed cell-type pair frequencies were compared to null distributions generated by 500 random permutations of cell-type labels (Supplementary Figure S4). Mouse-level enrichment z-scores were obtained by averaging across images per animal, and genotype comparisons were assessed via two-tailed Mann–Whitney U tests with Benjamini–Hochberg FDR correction (family size = 8).

**Figure 7:**
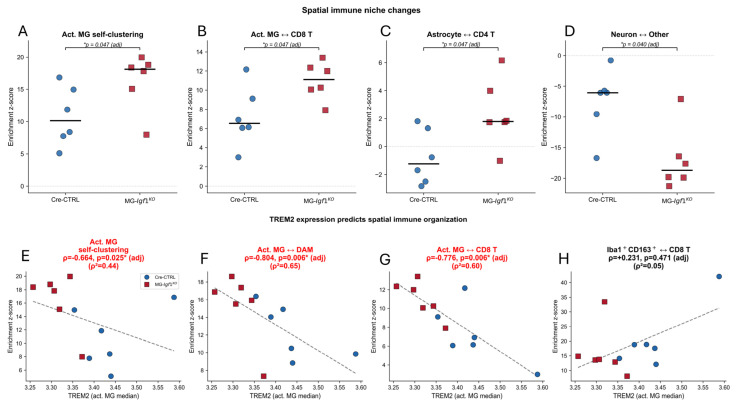
**Spatial immune niche reorganization and TREM2**–**spatial correlations in MG-*Igf1*^KO^ lesions.** (**A**–**D**) Mouse-level neighborhood enrichment z-scores for Cre-CTRL (blue circles, *n* = 6) and MG-*Igf1*^KO^ (red squares, *n* = 6) animals. For each tissue image, spatial neighbor graphs were constructed using a 30-pixel (30 µm) interaction radius and observed cell-type pair frequencies were compared to 500 random permutations of cell-type labels. Z-scores were averaged across images per mouse. (**A**) Activated microglia (Iba1^+^CD68^+^) self-clustering. (**B**) Activated microglia–CD8 T cell proximity. (**C**) Astrocyte–CD4 T cell enrichment. (**D**) Neuron–other cell spatial enrichment. Black bars indicate group medians. * *p*_adj values by two-tailed Mann–Whitney U test with Benjamini–Hochberg FDR correction (family size = 8). (**E**–**H**) Scatterplots of mouse-level TREM2 median intensity on activated microglia (*x*-axis) versus neighborhood enrichment z-scores (*y*-axis) for Cre-CTRL (blue circles) and MG-*Igf1*^KO^ (red squares). (**E**) Activated microglia self-clustering (ρ = −0.664, *p*_adj = 0.025). (**F**) Activated microglia–DAM proximity (ρ = −0.804, *p*_adj = 0.006). (**G**) Activated microglia–CD8 T cell proximity (ρ = −0.776, *p*_adj = 0.006). (**H**) Iba1^+^CD163^+^–CD8 T cell proximity (ρ = +0.231, *p*_adj = 0.471). Dashed lines indicate linear regression fits. Spearman ρ and BH FDR-adjusted *p*-values shown; ρ^2^ indicates variance explained. Three of four spatial metrics showed significant inverse correlations with TREM2 expression.

This analysis revealed coordinated spatial reorganization in MG-*Igf1*^KO^ lesions (Figure 7). Activated microglia (Iba1^+^CD68^+^) showed significantly increased self-clustering in MG-*Igf1*^KO^ animals (*p*_adj = 0.047; median *p* = 0.041 [0.026–0.093]; Figure 7A), accompanied by significantly increased activated microglia–CD8 T cell proximity (*p*_adj = 0.047; median *p* = 0.065; Figure 7B). Additional reorganization included altered astrocyte–CD4 T cell enrichment in MG-*Igf1*^KO^ (*p*_adj = 0.047; Figure 7C) and increased neuron–other cell exclusion (*p*_adj = 0.040; Figure 7D). Notably, all spatial genotype effects were consistent in direction across both high-demyelination and low-demyelination subgroups, indicating that the observed reorganization reflects genotype-driven changes in immune niche architecture rather than secondary effects of disease severity.

### 3.8. TREM2 Expression Associates with Spatial Immune Organization

To assess whether the microglial maturation defect was linked to spatial reorganization, we correlated mouse-level TREM2 expression on activated microglia with neighborhood enrichment z-scores across all 12 animals. TREM2 was inversely associated with activated microglia self-clustering (ρ = −0.664, *p* = 0.025 [adj]; Figure 7E) and showed a stronger inverse association with activated microglia–DAM co-localization (ρ = −0.804, *p* = 0.006 [adj]; Figure 7F), indicating that mice with the lowest TREM2 showed the greatest disruption of the activated-to-DAM spatial relationship. TREM2 expression was similarly inversely associated with activated microglia–CD8 T cell proximity (ρ = −0.776, *p* = 0.006 [adj]; Figure 7G). In contrast, Iba1^+^CD163^+^–CD8 T cell co-localization did not correlate with TREM2 (ρ = +0.231, *p* = 0.471; Figure 7H), suggesting that CD163^+^ spatial behavior is governed by factors beyond TREM2 maturation status.

Three of four spatial metrics showed significant inverse associations with TREM2 expression. While the moderate effect size in panel E (ρ^2^ = 0.44) reflects greater between-animal variability in activated microglia self-clustering than in the more tightly correlated DAM and CD8 T cell relationships (ρ^2^ = 0.65 and 0.60, respectively), the consistent inverse direction across three independent spatial metrics suggests that TREM2 maturation status scales with the integrity of the activated microglial niche. We acknowledge that these correlative findings cannot establish whether reduced TREM2 expression directly drives the observed spatial reorganization, or whether both reflect a shared upstream consequence of microglial IGF-1 loss. Future studies using TREM2 loss-of-function or TREM2–agonist intervention in the JHMV model will be required to resolve causality.

### 3.9. CD163^+^ Regulatory Myeloid Cells Disperse from CD8 T Cells and Lose Arginase1 at the Interface

**Figure 8:**
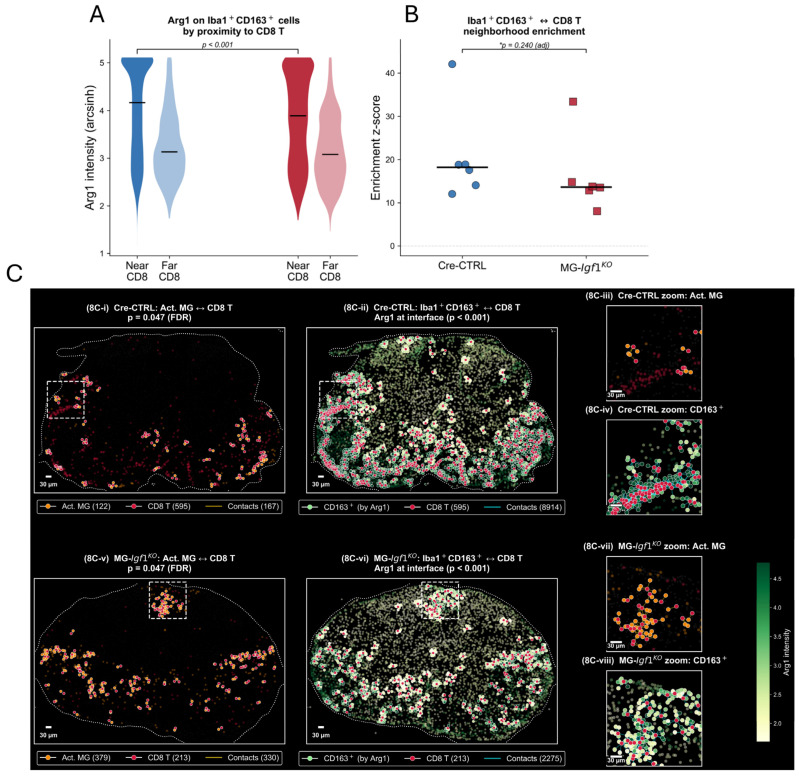
**CD163^+^ regulatory myeloid cells lose immunosuppressive capacity at the T cell interface.** (**A**) Mouse-level neighborhood enrichment z-scores for Iba1^+^CD163^+^–CD8 T cell spatial proximity in Cre-CTRL (blue circles, *n* = 6) and MG-*Igf1*^KO^ (red squares, *n* = 6) animals. Black bars indicate group medians. One Cre-CTRL animal showed an outlier z-score (42.3) reflecting unusually dense CD163^+^–CD8 clustering in a single ROI. (**B**) Arg1 expression on CD163^+^ cells stratified by proximity to CD8 T cells (near: ≤30 pixels, far: >30 pixels) across genotypes, * *p* ≤ 0.05. Violin plots show cell-level distributions; black bars indicate medians. In both genotypes, CD163^+^ cells near CD8 T cells express higher Arg1 than those farther away, but MG-*Igf1*^KO^ animals show significantly reduced Arg1 at the interface compared to Cre-CTRL. Statistical comparisons: (**A**) two-tailed Mann–Whitney U test on mouse-level z-scores; (**B**) cell-level comparison across ~16,000 CD163^+^ cells. This dual analysis reveals that CD163^+^ cells in MG-*Igf1*^KO^ lesions trend toward spatial dispersal from CD8 T cells and lose immunosuppressive capacity at remaining contact points. (**C**). **Spatial and functional reorganization of immune niches in** MG-*Igf1*^KO^ **lesions.** Representative tissue images showing spatial immune interactions in Cre-CTRL (top row) and MG-*Igf1*^KO^ (bottom row) spinal cord at 21 days post-JHMV infection. (**i**) Activated microglia (Iba1^+^CD68^+^, orange) and CD8 T cell (red) interactions within 30-pixel radius. Yellow lines connect interacting cell pairs. MG-*Igf1*^KO^ tissue shows increased clustering of activated microglia with CD8 T cells compared to controls. (**v**,**vii**). Iba1^+^CD163^+^ regulatory myeloid cells (colored by Arg1 intensity, see scale bar) and CD8 T cell (red) spatial relationships. Green coloring indicates high Arg1 expression; pale/white indicates low Arg1. In Cre-CTRL tissue (**ii**,**iv**), CD163^+^ cells near CD8 T cells display high Arg1 (bright green), consistent with immunosuppressive function at the regulatory interface. In MG-*Igf1*^KO^ tissue (**vi**,**vii**), CD163^+^ cells show markedly reduced Arg1 intensity at CD8 T cell contact points, indicating loss of regulatory capacity. Background cells (gray dots) show tissue architecture. Scale bars = 30 μm. This spatial analysis demonstrates the dual impairment in MG-*Igf1*^KO^ lesions: disrupted immune cell positioning and loss of functional regulatory capacity at remaining contact sites. Images selected from mouse C6M2 (Cre-CTRL) and C6M9 (MG-*Igf1*^KO^) representing median spatial enrichment values for each interaction type; both sections were from the same anatomical region.

Iba1^+^CD163^+^ regulatory myeloid cells (15,979 cells; 84.2% CX3CR1^+^) showed a trend toward reduced spatial enrichment with CD8 T cells in MG-*Igf1*^KO^ tissue (CN median z = 18.0, MG-*Igf1*^KO^ median z = 13.7; *p*_adj = 0.240; Figure 8A), consistent in direction across five of six MG-*Igf1*^KO^ mice. One Cre-CTRL mouse (C6M2) was a notable outlier (z = 42.3), reflecting an unusually dense CD163^+^–CD8 cluster in a single ROI; group-level results were unchanged with or without this animal. To assess the functional consequences of this spatial trend, we examined Arginase-1 (Arg1) expression on CD163^+^ cells as a function of their proximity to CD8 T cells. In Cre-CTRL animals, CD163^+^ cells near CD8 T cells (≤30 µm) expressed higher Arg1 than those farther away (near median: 4.17, far: 3.13), and the same proximity-dependent gradient was preserved in MG-*Igf1*^KO^ animals (near: 3.89, far: 3.13). However, the absolute Arg1 level on CD163^+^ cells near CD8 T cells was significantly lower in MG-*Igf1*^KO^ compared to Cre-CTRL (3.89 vs. 4.17; cell-level *p* < 0.001; Figure 8B), indicating that while MG-*Igf1*^KO^ myeloid cells still localize to CD8 T cell neighborhoods, they arrive with reduced immunosuppressive capacity. Importantly, bulk mouse-level analysis of marker expression on CD163^+^ cells revealed no significant differences for any of the 12 functional markers tested (all *p* > 0.39, Mann–Whitney U test), indicating that the Arg1 impairment is a spatially resolved phenomenon detectable only at the myeloid–T cell interface.

These spatial and functional changes are illustrated in representative tissue overlays derived from Hyperion CyTOF/IMC (Figure 8(C-i–viii)). In Cre-CTRL tissue, activated microglia (orange) form discrete clusters with CD8 T cells (red) connected by contact lines (Figure 8(C-i)), with the corresponding zoom revealing individual cell–cell contacts at the interface (Figure 8(C-iii)). CD163^+^ cells in the same Cre-CTRL region, colored by Arg1 intensity, show bright green clusters at CD8 T cell interfaces, reflecting high immunosuppressive capacity at regulatory contact points (Figure 8(C-ii), zoom in Figure 8(C-iv)). In MG-*Igf1*^KO^ tissue, activated microglia–CD8 T cell clustering is denser and more spatially concentrated (Figure 8(C-v), zoom in Figure 8(C-vii)), consistent with the significant enrichment detected by permutation testing. Critically, MG-*Igf1*^KO^ tissue shows both spatial dispersal of CD163^+^ cells from CD8 T cell neighborhoods and markedly reduced Arg1 intensity—visible as muted yellow-green rather than bright green—at the remaining contact sites (Figure 8(C-vi), zoom in Figure 8(C-viii)). These results argue for a dual impairment in MG-*Igf1*^KO^ lesions: CD163^+^ regulatory myeloid cells trend toward physical dispersal from CD8 T cells, and at the remaining points of contact, they show significantly reduced immunosuppressive capacity.

### 3.10. Spatial Immune Organization Associates with Clinical Disease Severity

**Figure 9:**
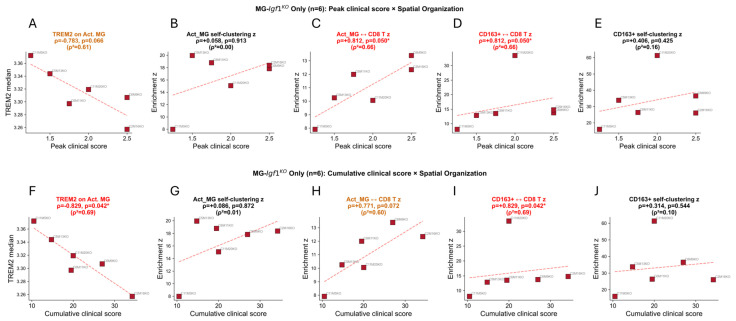
**Spatial immune disorganization correlates with clinical disease severity within MG-*Igf1*^KO^ animals.** Within-genotype Spearman correlations between spatial immune metrics and clinical disease scores in **MG-*Igf1*^KO^** animals only (*n* = 6). (**A**–**E**) Peak clinical score correlations. (**F**–**J**) Cumulative clinical score correlations. (**A**,**F**) TREM2 median intensity on activated microglia. (**B**,**G**) Activated microglia self-clustering z-score. (**C**,**H**) Activated microglia–CD8 T cell enrichment z-score. (**D**,**I**) Iba1^+^CD163^+^–CD8 T cell enrichment z-score. (**E**,**J**) Iba1^+^CD163^+^ self-clustering z-score. Dashed red lines indicate linear regression fits. Spearman ρ, uncorrected *p*-values, and ρ^2^ (variance explained) are shown per panel; asterisks (*) denote *p* < 0.05. Red title text indicates *p* < 0.05; orange indicates *p* < 0.10. These within-genotype correlations did not survive FDR correction for multiple comparisons (10-test family, all *p*_adj > 0.10), consistent with the limited statistical power of *n* = 6 within-genotype comparisons and should be considered exploratory. Effect sizes were large to very large for four correlations: Act_MG–CD8 × peak (ρ = +0.812, ρ^2^ = 0.66), CD163^+^–CD8 × peak (ρ = +0.812, ρ^2^ = 0.66), TREM2 × cumulative (ρ = −0.829, ρ^2^ = 0.69), and CD163^+^–CD8 × cumulative (ρ = +0.829, ρ^2^ = 0.69). Mouse IDs annotated for individual identification.

To assess whether immune spatial reorganization tracks with disease severity, we correlated mouse-level spatial metrics with clinical disease scores within MG-*Igf1*^KO^ animals (Figure 9). Among peak clinical score correlations (Figure 9A–E), TREM2 expression on activated microglia showed a trending inverse correlation (ρ = −0.783, *p* = 0.066; Figure 9A), suggesting that animals with lower microglial TREM2 developed more severe disease. Activated microglia self-clustering was not correlated with peak score (ρ = +0.058, *p* = 0.913; Figure 9B). In contrast, activated microglia–CD8 T cell proximity was positively correlated with peak score (ρ = +0.812, *p* = 0.050; Figure 9C), as was CD163^+^–CD8 T cell enrichment (ρ = +0.812, *p* = 0.050; Figure 9D), indicating that tighter myeloid–T cell spatial coupling predicted worse clinical outcomes. CD163^+^ self-clustering did not reach significance (ρ = +0.406, *p* = 0.425; Figure 9E).

Similar patterns emerged for cumulative clinical burden (Figure 9F–J). TREM2 expression was inversely correlated with cumulative score (ρ = −0.829, *p* = 0.042; Figure 9F), reaching nominal significance with this more integrated measure of disease severity. Activated microglia self-clustering was again not correlated (ρ = +0.086, *p* = 0.872; Figure 9G). Activated microglia–CD8 T cell proximity showed a trending association (ρ = +0.771, *p* = 0.072; Figure 9H). CD163^+^–CD8 T cell enrichment showed the strongest positive correlation of all metrics tested (ρ = +0.829, *p* = 0.042; Figure 9I), mirroring the peak score result and reinforcing that myeloid–T cell spatial proximity tracks with disease burden. CD163^+^ self-clustering was not correlated with cumulative score (ρ = +0.314, *p* = 0.544; Figure 9J).

These analyses are limited by the small MG-*Igf1*^KO^-only sample size (*n* = 6), and reported *p*-values are nominal rather than corrected for multiple comparisons. The consistent direction of effect across both peak and cumulative score measures, however, suggests that myeloid–T cell spatial coupling and TREM2 maturation track with the magnitude of clinical disease severity. Mechanistic confirmation will require larger cohorts and direct perturbation of the implicated spatial relationships.

## 4. Discussion

CyTOF analysis identified two reciprocal changes in T cell composition that together suggest microglial IGF-1 is required for maintaining the balance between effector and regulatory adaptive immunity in the demyelinated CNS. The increase in cluster abundance in MG-*Igf1*^KO^ mice was characterized as CD8^+^Ly6C^+^ T cells. Ly6C is a GPI-anchored surface glycoprotein that functions as an accessory molecule in CD8^+^ cytotoxic T lymphocyte-mediated cytolysis and lymphokine production [55]. On naïve CD8^+^ T cells, Ly6C expression is induced by type I interferon signaling and marks a subset with high self-reactivity that preferentially differentiates into terminal effector cells upon viral infection, with enhanced expansion and reduced memory cell generation [56]. Ly6C is also upregulated during CD8^+^ T cell differentiation into central memory cells, where it supports lymph node homing through LFA-1-dependent adhesion [57]. In the JHMV model, CD8^+^ T cells are the primary mediators of viral clearance through perforin-dependent cytolysis and IFN-γ secretion, but their sustained activation also contributes to immune-mediated demyelination [1]. The expansion of this CD8^+^Ly6C^+^ population in MG-*Igf1*^KO^ spinal cords is therefore consistent with a failure in immunoregulatory constraint, permitting accumulation of effector-poised cytotoxic T cells within the inflamed CNS.

The reciprocal loss of Tr1-like CD4^+^ T cells (provisional annotation) in MG-*Igf1*^KO^ mice reinforces this interpretation. While definitive Tr1 identification requires co-expression of CD49b and LAG-3 [54], the regulatory identity of this cluster is supported by its expression of CD39, an ectonucleotidase that generates immunosuppressive adenosine and is functionally required for Tr1-mediated suppression [58]. The selectivity of these changes is notable: of 19 immune clusters identified by unsupervised analysis, only these two populations were significantly altered in abundance, arguing against generalized inflammatory amplification and instead pointing to a specific failure of immunoregulatory constraint. This selective rebalancing, concurrent expansion of effector populations alongside contraction of regulatory and memory compartments, suggests that microglial IGF-1 loss plays a role in the disruption of the homeostatic balance between immune activation and resolution. The pattern of abundance changes implies that IGF-1 signaling supports coordinated behavior across the neuroimmune system, not simply individual cell types. This interpretation motivated our subsequent imaging mass cytometry analysis, which asked whether disrupted population-level coordination is reflected in spatial reorganization of immune niches within tissue.

We also identified three convergent spatial phenotypes linking microglial IGF-1 to neuroimmune regulation during viral-induced demyelination: [1] a TREM2 maturation block on activated microglia, ref. [2] spatial dispersal of CD163^+^ regulatory myeloid cells from CD8 T cell niches, and [3] loss of Arg1 expression at the myeloid–T cell interface. These defects appear mechanistically linked: impaired TREM2-dependent microglial maturation propagates into tissue-level immune niche reorganization, in which maturation-blocked activated microglia form dense inflammatory clusters with CD8 T cells while CD163^+^ regulatory myeloid cells disperse and lose immunosuppressive capacity at the remaining contact points. Within MG-*Igf1*^KO^ animals, the degree of spatial disruption correlates with clinical disease severity, linking immune niche architecture to functional outcomes. These spatial relationships, undetectable by conventional flow cytometry, extend our CyTOF findings and demonstrate that IGF-1 signaling in microglia is required not only for intrinsic microglial activation but for maintaining the spatial architecture of immunoregulatory circuits in the inflamed CNS.

### 4.1. IGF-1 Deletion Associates with TREM2-Dependent Microglial Maturation

An important finding in our spatial dataset was the selective reduction in TREM2 protein on activated microglia in MG-*Igf1*^KO^ tissue, compared to CRE-CTRL. TREM2 is a well-established regulator of microglial activation during demyelination. In the cuprizone model, TREM2-deficient microglia fail to upregulate phagocytic receptors, lipid catabolism genes, and trophic factors—including IGF-1 itself—resulting in impaired myelin debris clearance and persistent demyelination [59,60]. Conversely, agonistic TREM2 antibodies enhance myelin uptake, accelerate debris removal, and increase oligodendrocyte precursor density in a dose-sensitive manner [61]. Our data are consistent with a bidirectional relationship between TREM2 and IGF1 signaling; while TREM2 drives IGF-1 expression in activated microglia, IGF-1 signaling appears required for sustained TREM2 upregulation during the phagocytic response. Definitive testing of this hypothesis will require direct manipulation of both pathways.

This interpretation is supported by recent single-cell RNA sequencing data from ischemic brain tissue, in which microglia co-expressing high levels of both IGF-1 and TREM2 were identified as a neuroprotective population, and replenishment of these cells reversed ischemic injury via a proposed TREM2–IGF-1 signaling axis [62]. A similar reciprocal relationship has been described in aged *Trem2*^−^/^−^ mice, where corpus callosum microglia exhibit both reduced numbers and abnormal morphology [59], consistent with the activated-but-immature phenotype we observe in MG-*Igf1*^KO^ tissue.

Within the conserved macrophage activation framework described by Sanin et al. [63], TREM2 function maps onto the phagocytic regulatory trajectory, along which macrophages commit to tissue homeostasis through expression of Mrc1, CD163, and MerTK. Disruption of progression along this path—whether through genetic ablation or stalling—has pathological consequences across disease contexts, including atherosclerosis and neurodegeneration [63]. The selective association of IGF-1 deletion with reduced TREM2 on activated microglia is consistent with a possible cell-intrinsic role for IGF-1 in maturation along this phagocytic regulatory trajectory, although the current correlative findings cannot establish this directly.

### 4.2. CD163^+^ Regulatory Myeloid Cells and Spatial Reorganization

The CD163^+^Iba1^+^ population identified in our spatial analysis represents a regulatory myeloid subset distinct from homeostatic microglia. CD163 is a scavenger receptor for haptoglobin–hemoglobin complexes, induced by anti-inflammatory cytokines IL-10 and IL-6 and associated with the resolution phase of inflammation [64]. In the healthy CNS, CD163 expression is restricted to perivascular macrophages; parenchymal CD163^+^ cells appear only during active neuroinflammation and are thought to derive from infiltrating monocytes or represent a disease-associated microglial state [65]. In MS lesions, parenchymal CD163^+^ macrophages co-express MHC-II and contain myelin basic protein, suggesting active antigen processing, yet display an anti-inflammatory profile consistent with the resolution of inflammation [66]. Soluble CD163 is elevated in the CSF of MS patients, indicating local CNS production during demyelinating disease [67].

In our model, CD163^+^ regulatory myeloid cells showed reduced spatial enrichment with CD8 T cells in MG-*Igf1*^KO^ tissue, a trend consistent across most infected mice. While the group-level comparison did not reach significance, the functional significance of this spatial change was supported by orthogonal analyses. Within MG-*Igf1*^KO^ animals, higher CD163^+^–CD8 spatial proximity correlated with worse clinical outcomes, suggesting that CD163^+^ cells remaining at T cell interfaces had lost functional competence, consistent with the Arg1 reduction observed at these contact points. This spatial reorganization aligns with the Sanin et al. [63], in which CD163 marks macrophages along the phagocytic regulatory activation trajectory. The simultaneous aggregation of inflammatory activated microglia and dispersal of CD163^+^ regulatory cells in MG-*Igf1*^KO^ spinal cords suggests that IGF-1 deletion does not simply impair one myeloid population but shifts the balance between pro-inflammatory and resolution-competent myeloid programs. Whether CD163^+^ cells require direct IGF-1 signaling or are displaced secondarily by the inflammatory microenvironment created by IGF-1-deficient microglia remains to be determined.

A robust spatial finding was the significant loss of Arg1 expression on Iba1^+^ myeloid cells positioned within CD8 T cell neighborhoods in MG-*Igf1*^KO^ tissue. Arginase-1 competes with inducible nitric oxide synthase for L-arginine; by depleting extracellular arginine, Arg1-expressing myeloid cells suppress T cell proliferation and cytokine production through downregulation of the CD3ζ chain [68]. This mechanism is recognized as a fundamental pathway of inflammation-associated immunosuppression across disease contexts [69].

In the CNS, CD163 expression is normally restricted to perivascular macrophages; however, during viral encephalitis, resident microglia can upregulate CD163 as part of their activation response [65,70]. This virus-induced CD163 expression has been demonstrated across multiple CNS viral infections and correlates with microglial activation and viral replication. Myeloid-specific Arg1 deletion worsens neuronal loss in retinal ischemia [71] and exacerbates amyloid pathology in Alzheimer’s disease models [72], establishing myeloid Arg1 as neuroprotective in CNS injury. Transcriptomic analysis further revealed that myeloid Arg1 insufficiency shifts microglia toward a homeostatic, non-phagocytic signature during amyloidosis, impairing Aβ clearance [63], establishing myeloid Arg1 as neuroprotective in CNS injury. In the tumor microenvironment, macrophage Arg1 directly suppresses CD8^+^ T cell infiltration and function; genetic or pharmacological Arg1 inhibition increases CD8 T cell numbers and sensitizes tumors to immune checkpoint blockade [73].

Our spatial data reveal that this Arg1-mediated immunoregulatory axis operates at the specific interface where virus-activated CD163^+^ microglia/myeloid cells encounter CD8 T cells. The loss of Arg1 at this interface in MG-*Igf1*^KO^ tissue suggests that in the absence of microglial IGF-1 signaling, activated microglia fail to establish the arginine-depleted microenvironment necessary for local T cell suppression. Recent work has demonstrated that high arginase activity in myeloid cells depletes extracellular arginine, inducing T cell hyporesponsiveness that is reversible upon arginine restoration [74]. The spatial specificity of our finding that Arg1 loss is concentrated at the T cell interface rather than across all myeloid cells implies that this is not a generalized failure of myeloid polarization but a disruption of the immunoregulatory niche that normally restrains adaptive immunity in the demyelinated CNS.

### 4.3. An Integrated Model: IGF-1 as a Regulator of Neuroimmune Spatial Architecture

Taken together, our findings are consistent with a model in which microglial IGF-1 coordinates multiple aspects of the myeloid regulatory response to demyelination. In control tissue, microglia respond to myelin damage by upregulating TREM2, which drives phagocytic maturation, lipid processing, and trophic factor production [59]. This microglial activation appears to support the positioning of CD163^+^ regulatory myeloid cells at the adaptive immune interface, where Arg1 expression creates a locally immunosuppressive environment that restrains CD8 T cell effector function. In the absence of microglial IGF-1, this coordinated response is disrupted at multiple levels. TREM2 maturation stalls, impairing the phagocytic program that normally resolves myelin debris and contributes to anti-inflammatory signaling. CD163^+^ regulatory cells disperse from CD8 T cell niches, possibly reflecting the loss of IGF-1-dependent recruitment signals or impaired retention cues from TREM2-deficient microglia. Arg1 is lost at the myeloid–T cell interface, removing a putative metabolic brake on T cell activation. The net result is a spatial environment that may favor unchecked inflammatory signaling in the demyelinated spinal cord.

CD8 T cells in the CNS do not primarily engage neurons or oligodendrocytes directly; rather, immunohistochemical studies in MS have demonstrated that CD8 T cells predominantly interact with CD11b^+^ myeloid cells, including microglia, with consequences including myeloid ROS production, pro-inflammatory cytokine release, and collateral neuronal damage [75,76]. TREM2 expression on microglia is associated with regulatory functions including enhanced phagocytosis and reduced pro-inflammatory cytokine production [54,55]. The convergence of TREM2 loss, CD163^+^ dispersal, and Arg1 depletion thus describes a multi-layered failure of the myeloid regulatory programs that normally contain adaptive immune responses within the CNS. This model is supported by our clinical correlation data, in which spatial immune disorganization tracked with disease severity within MG-*Igf1*^KO^ animals: the most severely affected mice showed both spatial retention of dysfunctional CD163^+^ cells and characteristic Arg1 reduction at the T cell interface.

### 4.4. Perspectives

Using the JHMV model of neuroinflammation and demyelination, we have demonstrated the microglia-derived IGF-1 does not impact innate and adaptive immune responses associated with the control of viral replication during acute disease. However, during chronic disease, JHMV-infected MG-*Igf1*^KO^ mice exhibited worsened clinical disease that was associated with an increase in the severity of spinal cord demyelination. Through use of imaging mass cytometry, we have identified a candidate axis linking IGF-1 signaling and microglial function in the JHMV model of viral-induced neurologic disease. The most significant marker-level change in MG-*Igf1*^KO^ mice was the reduction in TREM2 expression on activated microglia that associated with impaired transition to the DAM phenotype. Given that the DAM program is essential for sensing damage and clearing debris, this maturation defect may contribute to the exacerbated white matter damage observed in MG-*Igf1*^KO^ mice. Further, these findings also argue that microglia-derived IGF-1 supports the coordinated behavior of myeloid and T cell populations and may help organize the spatial immune niche in ways that limit excessive T cell-mediated damage. Mechanistic confirmation of these relationships will require targeted perturbation of the implicated pathways in future studies.

## Data Availability

The data presented in this study and analysis code are available from the corresponding author upon reasonable request.

## Supplementary Materials

The following supporting information can be downloaded at: https://www.mdpi.com/article/10.3390/v18050550/s1, Figure S1. opt-SNE visualization of cluster overlays for all immune cell clusters by genotype; Figure S2. opt-SNE visualization of all immune cell clusters by genotype; Figure S3. CyTOF cluster marker expression profile. Figure S4. Neighborhood enrichment analysis of spatial immune organization in Cre-CTRL and MG-Igf1KO spinal cords; Table S1. Mass cytometry (CyTOF) antibody panel; Table S2. Key immune populations identified by CyTOF mass cytometry; Table S3. Imaging mass cytometry (IMC) antibody panel.

## Abbreviations

The following abbreviations are used in this manuscript:

IGF-1	Insulin growth factor 1
JHMV	Glial-tropic JHM strain of mouse hepatitis virus
DAM	Disease-associated microglia
CNS	Central nervous system
ASC	Antibody-secreting cells
Tregs	T regulatory cells
OPC	Oligodendrocyte progenitor cells
PFU	Plaque-forming unit
OCT	Optimum cutting temperature
LFB	Luxol fast blue
MOG	Myelin oligodendrocyte glycoprotein
CyTOF	Cytometry time-of-flight
CAS	Cell acquisition solution
KO	Knockout (MG-*Igf1*^KO^)
CN	Control (Cre-CTRL)
NaN	Not a number
IMC	Imaging mass cytometry
SAM	Segment anything model
ROI	Region of interest
HPC	High-performance computing
GPU	Graphics processing unit
FDR	False discovery rate
SOM	Self-organizing map
DMIR	Differential Intensity Map-based Restoration
DeepSNIF	Deep learning-based Short Noise image Filtering
EAE	Experimental autoimmune encephalomyelitis
GAPDH	Glyceraldehyde-3-Phosphate Dehydrogenase
opt-SNE	Optimized t-distributed stochastic neighbor embedding
IGF-1R	Insulin growth factor 1 receptor
