# Supplementary material for: Microglial-Derived IGF-1 Serves as a Regulator for Neuroimmune Homeostasis During Viral-Induced Demyelination
Source: Viruses. 2026 May 9;18(5):550. doi: 10.3390/v18050550 (PMC13211618; doi:10.3390/v18050550)
Supplement: Supplementary file 1 [file viruses-18-00550-s001.zip › viruses-4272250-supplementary.pdf]

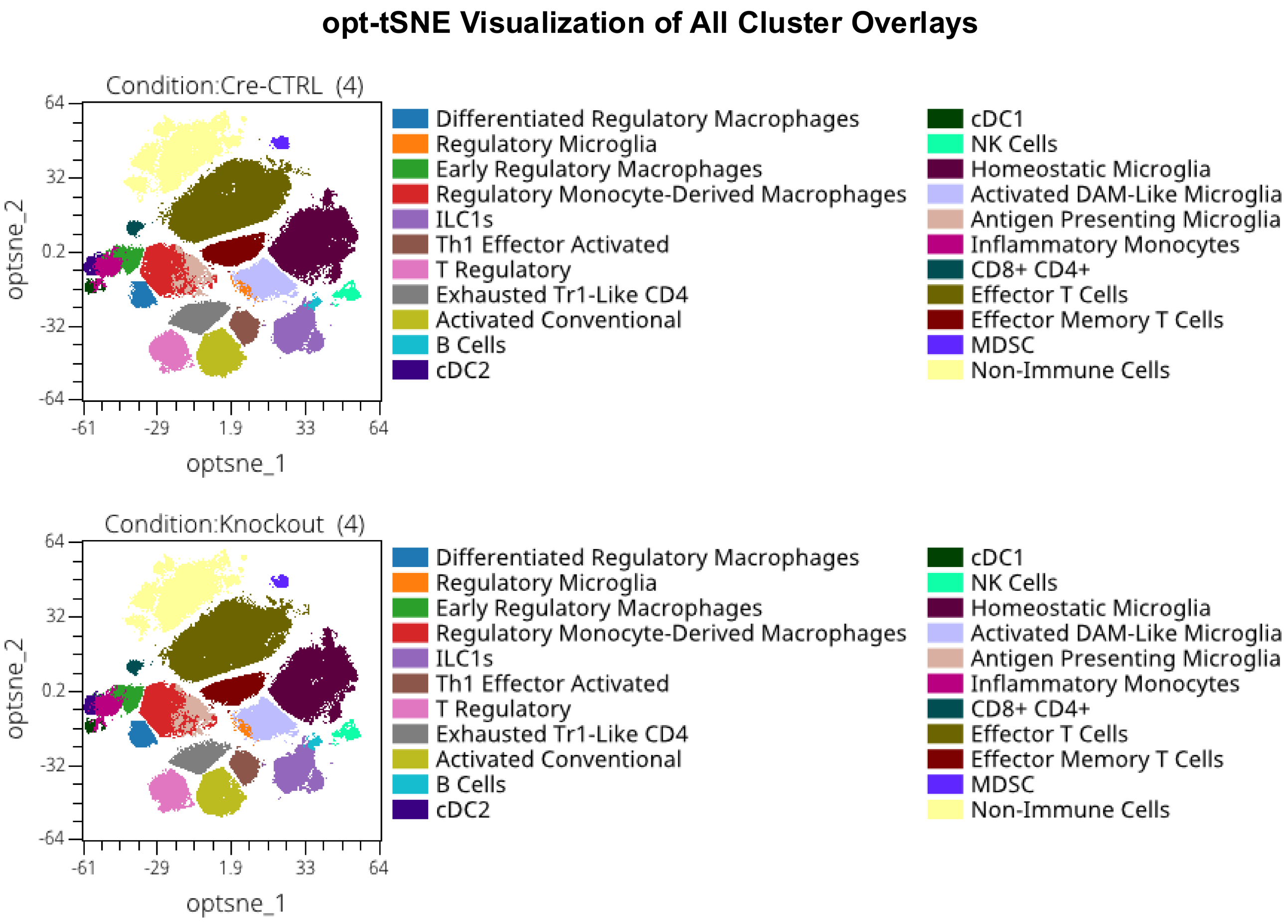

Supplementary Figure S1. opt-SNE visualization of cluster overlays for all immune cell clusters by genotype. Optimized t-SNE (opt-SNE) projections of 62,204 cells from the 36-marker IMC panel, split by condition: Cre-CTRL (top, n=4 mice shown) and MG-Igf1^{KO} (bottom, n=4 mice shown). Cells are colored by cluster identity across 23 populations identified by unsupervised FlowSOM clustering and manual annotation. Clusters span myeloid lineages (Differentiated Regulatory Macrophages, Early Regulatory Macrophages, Regulatory Monocyte-Derived Macrophages, Inflammatory Monocytes, MDSC, cDC1, cDC2), microglial subsets (Regulatory Microglia, Homeostatic Microglia, Activated DAM-Like Microglia, Antigen Presenting Microglia), lymphoid populations (Effector T Cells, Effector Memory T Cells, $CD8^{+}CD4^{+}$, Th1 Effector Activated, T Regulatory, Exhausted Tr1-Like CD4, Activated Conventional, B Cells, NK Cells, ILC1s), and Non-Immune Cells. Overall cluster architecture is preserved between genotypes, with the two populations showing significant differential abundance — $CD8^{+}Ly6C^{+}$ effector T cells (expanded in KO) and Exhausted Tr1-Like $CD4^{+}$ T cells (reduced in KO).

opt-tSNE Visualization of Marker Expression

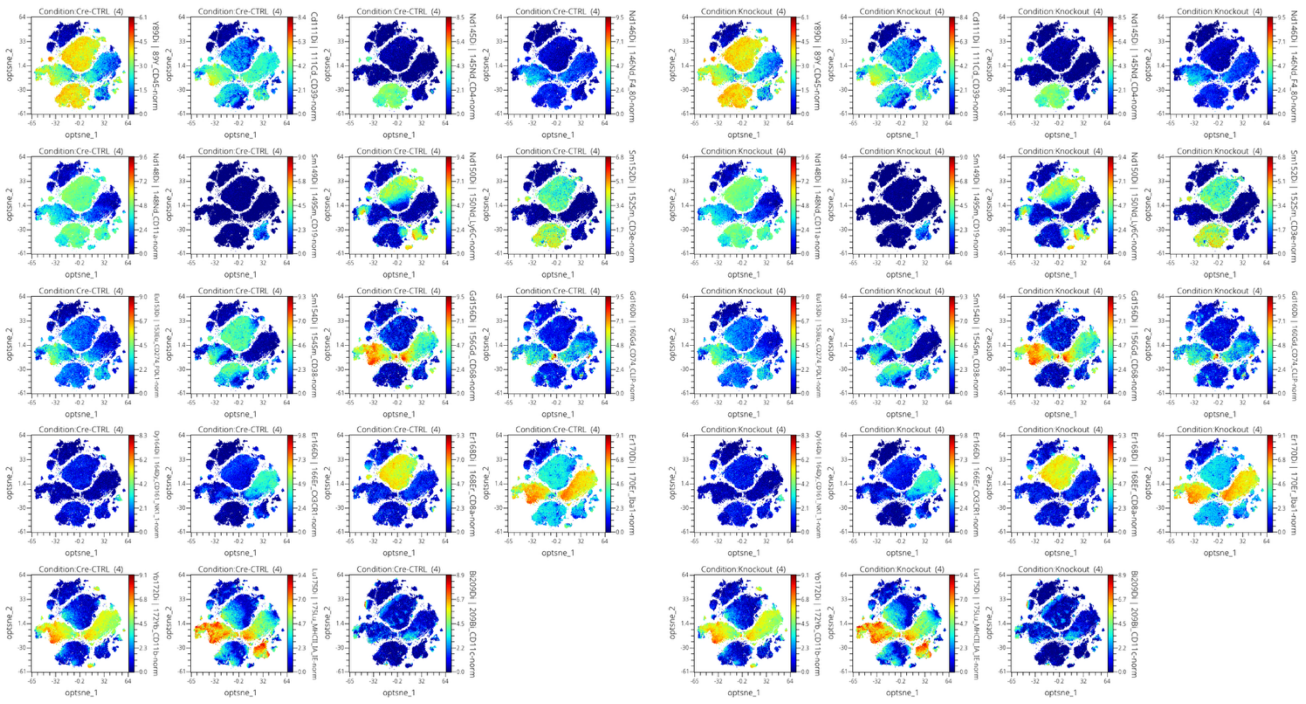

Supplementary Figure S2. **opt-SNE visualization of all immune cell clusters by genotype.** Optimized t-SNE (opt-SNE) projections of CyTOF mass cytometry data, split by condition: Cre-CTRL (top, n=4) and MG-Igf1^{KO} ("Knockout", n=4). Cells are colored by cluster identity used to phenotype immune populations identified by unsupervised FlowSOM clustering (25 metaclusters, 2 non-immune excluded) and manual annotation based on median marker expression profiles. Clusters span microglial subsets (Homeostatic, Regulatory, Activated DAM-Like, Antigen Presenting Microglia), macrophage/myeloid populations (Early Regulatory, Differentiated Regulatory, Regulatory Monocyte-Derived Macrophages, Inflammatory Monocytes, MDSC), dendritic cells (cDC1, cDC2), T cell subsets (Effector T Cells, Effector Memory T Cells, Th1 Effector Activated, Activated Conventional, T Regulatory, Exhausted Tr1-Like CD4), and other lymphoid cells (NK Cells, B Cells). Overall cluster architecture is preserved between genotypes. Data were arcsinh-transformed (cofactor=5) prior to dimensionality reduction.

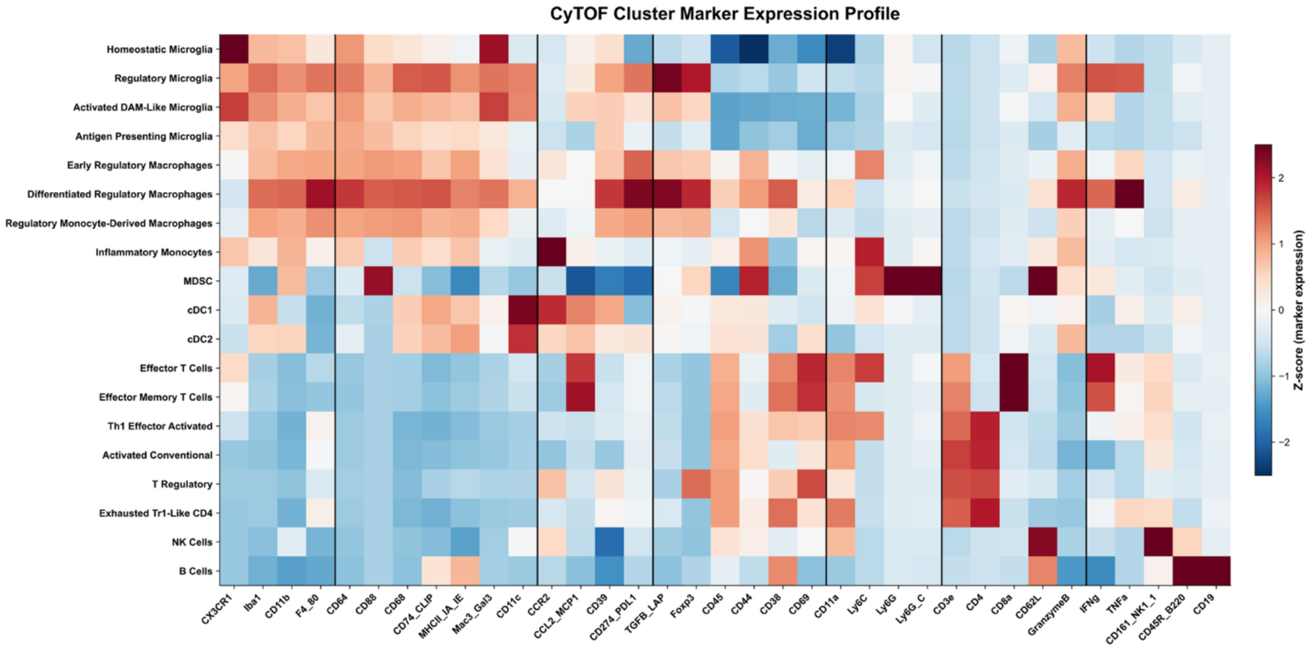

Supplementary Figure S3. CyTOF cluster marker expression profile. Heatmap showing the relative expression of 35 surface and intracellular markers across 19 immune cell populations identified by FlowSOM unsupervised clustering and manual annotation of CyTOF data. Values are z-scored per marker (column) across all clusters to highlight relative enrichment (red) or depletion (blue) of each marker within each population. Marker columns are organized by functional category from left to right: microglial identity (CX3CR1, Iba1), myeloid lineage (CD11b, F4/80, CD64, CD88, CD68), antigen presentation/myeloid function (CD74/CLIP, MHC-II, Mac-3/Gal-3, CD11c), migration (CCR2, CCL2/MCP-1), regulatory/checkpoint (CD39, PD-L1, TGFβ-LAP, Foxp3), activation/differentiation (CD45, CD44, CD38, CD69, CD11a), granulocyte/monocyte markers (Ly6C, Ly6G, Ly6G/C), T cell lineage (CD3e, CD4, CD8a), T cell homing (CD62L), effector molecules (Granzyme B, IFN-γ, TNF-α), and lymphoid identity (NK1.1, B220, CD19). Vertical black lines delineate marker functional groups. Cluster rows are ordered by cell lineage: microglia (Homeostatic, Regulatory, Activated DAM-Like, Antigen Presenting), macrophages/myeloid (Early Regulatory, Differentiated Regulatory, Regulatory Monocyte-Derived, Inflammatory Monocytes, MDSC), dendritic cells (cDC1, cDC2), T cells (Effector, Effector Memory, Th1 Effector Activated, Activated Conventional, T Regulatory, Exhausted Tr1-Like CD4), and other lymphoid (NK Cells, B Cells). Markers are ordered to produce a diagonal pattern in which defining markers align with their corresponding cluster positions, facilitating visual assessment of cluster annotation quality. Expression values represent the mean of Cre-CTRL and MG-Igf1^{KO} conditions, as this figure illustrates cluster identity rather than genotype-specific differences. Data were arcsinh-transformed (cofactor=5) prior to analysis.

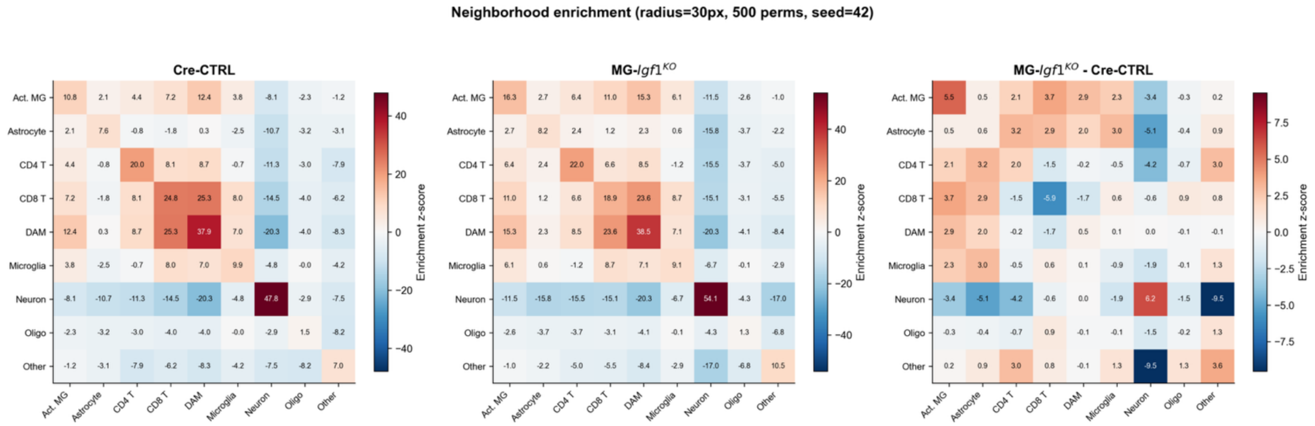

Supplementary Figure S4. Neighborhood enrichment analysis of spatial immune organization in Cre-CTRL and MG-Igf1^{KO} spinal cords. Pairwise spatial enrichment z-score matrices for 9 cell-type clusters identified by IMC, computed using permutation-based neighborhood analysis (30 μm interaction radius, 500 permutations, seed=42). (Left) Cre-CTRL group mean enrichment matrix (n=6). (Center) MG-Igf1^{KO} group mean enrichment matrix (n=6). (Right) Difference matrix (KO − CTRL), highlighting spatial interactions that changed between genotypes. Positive z-scores (red) indicate spatial co-enrichment (cell types found near each other more than expected by chance); negative z-scores (blue) indicate spatial avoidance. Values in each cell represent the mean enrichment z-score across all mice within the group. Notable changes include increased activated microglia self-clustering (+5.5) and activated microglia–CD8 T cell proximity (+3.7) in KO, alongside increased neuron–other cell avoidance (−9.5). Mouse-level z-scores were obtained by averaging across tissue images per animal; images with fewer than 50 cells were excluded. Cell types: Act. MG = activated microglia (Iba1^{+}CD68^{+}), Astrocyte, CD4 T, CD8 T, DAM = disease-associated microglia, Microglia = homeostatic microglia, Neuron, Oligo = oligodendrocytes, Other = unclassified cells.

Mass Cytometry Antibody Panel

Vendor	Catalog #	Target	Clone	Mass	Element	Channel	Conc (μg/mL)
Biolegend	103102	CD45	30-F11	89	Y	89Y	1
Biolegend	103002	CD44	IM7	106	Cd	106Cd	2
Life Technologies	14-0391-82	CD39	24DMS1	111	Cd	111Cd	4
Biolegend	139302	CD64	X54-5/7.1	113	Cd	113Cd	4
Biolegend	135802	CD88	20/70	116	Cd	116Cd	4
Biolegend	127637	Ly6G	1A8	141	Pr	141Pr	2
Biolegend	108402	Ly6-C/G	RB6-8C5	142	Nd	142Pr	2
Biolegend	104533	CD69	H1.2F3	143	Nd	143Nd	4
Biolegend	100506	CD4	RM4-5	145	Nd	145Nd	4
Biolegend	123143	F4/80	BM8	146	Nd	146Nd	4
Biolegend	101101	CD11a	M17/4	148	Nd	148Nd	2
Biolegend	115547	CD19	6D5	149	Sm	149Sm	2
Biolegend	138039	Ly6C	HK.14	150	Nd	150Nd	2
Biolegend	104443	CD62L	MEL-14	151	Eu	151Eu	4
Biolegend	100342	CD3ε	145-2C11	152	Sm	152Sm	4
Biolegend	124301	CD274_PDL1	10F.9G2	153	Eu	153Eu	4
Biolegend	102702	CD38	90	154	Sm	154Sm	4
Biolegend	141402	TGFRB1_LAP	TW7-16B4	155	Gd	155Gd	1
Biolegend	137001	CD68	FA-11	156	Gd	156Gd	1
Invitrogen	14-5773-82	Foxp3	FJK-16s	158	Gd	158Gd	1
Biolegend	326802	CD74_CLIP	In1/CD74	160	Gd	160Gd	4
Biolegend	103202	B220_CD45R	RA36B2	162	Dy	161Dy	2
Biolegend	108702	NK1.1	PK136	164	Dy	164Dy	4
Biolegend	341602	CX3CR1	Polyclonal Goat IgG	166	Er	166Er	4
Invitrogen	MA5-17040	CCL2	2D8	167	Er	167Er	1
Biolegend	100755	CD8a	53-6.7	168	Er	168Er	2
Wako	019-19741	Iba1	polyclonal	170	Er	170Er	1
Cedarlane	019-19741	Mac2-Gal3	M3/38	171	Yb	171Yb	4
Biolegend	101202	CD11b_Mac_1	M1/70	172	Yb	172Yb	2
Biolegend	662802	Granzyme B	12F9B65	173	Yb	173Yb	1
NovusBio	NBP1-48338	CCR2	Polyclonal	174	Yb	174Yb	4
Standard Bio Tools	3174003B	I-A/I-E	MS/114.15.2	175	Lu	175Lu	4
Standard Bio Tools	3209005B	CD11c	N418	209	Bi	209Bi	4
—	—	DNA1 (Ir191)	—	191	Ir	191Ir	1:400
—	—	DNA2 (Ir193)	—	193	Ir	193Ir	1:400

Supplementary Table S1. Mass cytometry (CyTOF) antibody panel. A 41-marker metal-conjugated antibody panel was applied to single-cell suspensions isolated by Percoll gradient from murine spinal cords of Cre-CTRL and MG-*Igf1*^{KO} mice (n = 4 per group) at 21 days post-JHMV infection. All antibodies were provided by the UC Irvine Stem Cell Research Center Flow & Mass Cytometry Core antibody bank and conjugated using Standard BioTools Maxpar X8 and MCP9 (cadmium) metal conjugation kits according to the manufacturer's protocol. Cells were stained sequentially for viability (cisplatin), surface antigens, and cytoplasmic/secreted proteins, followed by fixation and Cell-ID Intercalator-IR (Ir191/Ir193) staining for nuclear identification, per Standard BioTools' Maxpar Cytoplasmic/Secreted Antigen Staining with Fresh Fix protocol. Samples were acquired on a Standard BioTools Helios mass cytometer at a target concentration of 1.0 × 10^6 cells/mL (300–500 events/s). FCS files were normalized using CyTOF Software

v7.0.8493 and analyzed in OMIQ (Dotmatics) with batch correction by CytoNorm and unsupervised clustering by FlowSOM (21 metaclusters).

Key Immune Populations

Cluster	Lineage	Key Markers	Functional Annotation
**Effector $CD8^+$ T Cells**	$CD8^+$ T Cell	$CD8^+$ $Ly6C^+$ $CD44^+$ $CD11a^+$ $CD38^{med}$	IFN-I driven terminal effector; enhanced cytotoxicity
**Exhausted Tr1-Like $CD4^+$**	$CD4^+$ T Cell	Foxp3– Ly6C– $CD38^+$ $CD39^+$ $MHCII^{lo}$	Suppressive or dysfunctional; IL-10 producing (if Tr1)
**Early Regulatory Macrophages**	Monocyte-Derived	$CD45^{med}$ $Ly6C^{med/hi}$ PD-$L1^+$ $CD39^{med}$ $CD68^{hi}$ $MHCII^{med/hi}$	Transitioning regulatory monocyte-derived macrophage
**Differentiated Regulatory Macrophages**	Monocyte-Derived	$CD45^{hi}$ Ly6C– $CD64^{med}$ PD-$L1^+$ $CD39^{hi}$ $CD68^{hi}$ $MHCII^{hi}$	Maximally suppressive ($CD39^{hi}$); tissue-adapted regulatory
**Inflammatory Monocytes**	Monocyte	$CD45^{hi}$ $Ly6C^{hi}$ $CD64^+$ PD-$L1^{lo}$ $CD39^{lo}$ $MHCII^{hi}$	Pro-inflammatory; recruitment and amplification

Supplementary Table S2. Key immune populations identified by CyTOF mass cytometry. Summary of the five immune cell clustersshowing the most biologically significant changes or functional relevance in MG-$Igf1^{KO}$ spinal cords at 21 days post-JHMV infection. Clusters were identified by FlowSOM unsupervised clustering and manually annotated based on median marker expression profiles from a 41-marker CyTOF panel. Columns indicate cluster name, cell lineage of origin, defining surface marker phenotype (superscripts denote relative expression intensity: hi = high, med = medium, lo = low, + = positive, − = negative), and proposed functional annotation based on marker expression patterns and published literature. Effector $CD8^+$ T cluster abundance were significantly expanded and Exhausted Tr1-Like $CD4^+$ T cluster abundance were significantly reduced in MG-$Igf1^{KO}$ mice (n=4 per group). The three myeloid populations represent stages along the monocyte-to-regulatory macrophage differentiation trajectory, with progressive downregulation of Ly6C and upregulation of CD39 indicating increasing immunosuppressive capacity. Full marker expression profiles across all 21 immune clusters are shown in Supplementary Figure S2.

IMC Antibody Panel and Concentrations

Vendor	Catalog #	Target	Clone	Mass	Element	Channel	Conc (µg/mL)
Biolegend	103102	**CD45**	30F11	141	Pr	141Pr	8
Abcam	ab215989	**S100b**	EPY1576Y	143	Nd	143Nd	2
Biolegend	103002	**CD44**	IM7	144	Nd	144Nd	4
Invitrogen	MA544519	**CD4**	BLR167J	145	Nd	145Nd	8
Abcam	ab303566	**CD68**	RM1031	148	Nd	148Nd	5
Biolegend	644702	**GFAP**	2E1.E9	149	Sm	149Sm	2
Abcam	ab236033	**MAP2**	EPR19691	151	Eu	151Eu	2
Thermo Fisher	14-9985-82	**PD-1**	J43	152	Sm	152Sm	5
CST	25514SF	**F4/80**	D2S9R	154	Sm	154Sm	8
Abcam	ab213612	**CD163**	EPR19518	155	Gd	155Gd	6
Thermo Fisher	MM700	**IFNγ**	XMG1.2	156	Gd	156Gd	6
Abcam	ab250715	**MerTK**	EPR17534-139	158	Gd	158Gd	6
ProteinTech	11114-1-AP	**TAP1**	Polyclonal	160	Gd	160Gd	5
Abcam	ab278046	**LAMP1**	EPR21026	161	Dy	161Dy	6
Abcam	ab307165	**TNFα**	RM1005	162	Dy	162Dy	6
Abcam	ab300498	**Clec7a**	EPR25360	163	Dy	163Dy	8
R&D Systems	AF5868	**Arg1**	Polyclonal	164	Dy	164Dy	3
Abcam	ab251607	**CD3e**	CAL57	165	Ho	165Ho	6
R&D Systems	AF5825	**CX3CR1**	Polyclonal	166	Er	166Er	5
Abcam	ab245999	**APOE**	EPR19378	167	Er	167Er	5
Invitrogen	14-0195-82	**CD8a**	4SM16	168	Er	168Er	8
Wako	019-19741	**Iba1**	Polyclonal	170	Er	170Er	8
Cedarlane	CL8942AP	**Mac2/Gal3**	M3/38	171	Yb	171Yb	6
Abcam	ab209970	**CD11b**	EPR1344	172	Yb	172Yb	5
Abcam	ab80276	**PD-L1**	MIH6	173	Yb	173Yb	5
Abcam	ab240558	**CD11c**	EPR21826	175	Lu	175Lu	6
R&D Systems	AF1729	**TREM2**	Polyclonal	176	Yb	176Yb	5
—	—	**DNA1 (Ir191)**	—	191	Ir	191Ir	—
—	—	**DNA2 (Ir193)**	—	193	Ir	193Ir	—

Supplementary Table S3. Imaging mass cytometry (IMC) antibody panel. A 27-marker metal-conjugated antibody panel was applied to murine spinal cord tissue sections from Cre-CTRL and MG-*Igf1*^{KO} mice (n = 6 per group) at 21 days post-JHMV infection. All antibodies were conjugated to lanthanide metals using Standard BioTools Maxpar X8 metal conjugation kits. Final concentrations (µg/mL) represent the working concentration in the antibody cocktail. Ir191/Ir193 intercalator channels were used for nuclear identification.
